# Circulating Exosomal miRNA Profile During Term and Preterm Birth Pregnancies: A Longitudinal Study

**DOI:** 10.1210/en.2018-00836

**Published:** 2018-10-24

**Authors:** Ramkumar Menon, Chirantan Debnath, Andrew Lai, Dominic Guanzon, Shinjini Bhatnagar, Pallavi K Kshetrapal, Samantha Sheller-Miller, Carlos Salomon, The Garbhini Study Team

**Affiliations:** 1 Division of Maternal-Fetal Medicine and Perinatal Research, Department of Obstetrics and Gynecology, University of Texas Medical Branch at Galveston, Galveston, Texas; 2 Translational Health Science and Technology Institute, Faridabad, Haryana, India; 3 Exosome Biology Laboratory, Centre for Clinical Diagnostics, University of Queensland Centre for Clinical Research, Royal Brisbane and Women’s Hospital, University of Queensland, Brisbane, Queensland, Australia; 4 Department of Clinical Biochemistry and Immunology, Faculty of Pharmacy, University of Concepción, Concepción, Chile

## Abstract

Despite decades of research in the field of human reproduction, the mechanisms responsible for human parturition still remain elusive. The objective of this study was to describe the changes in the exosomal miRNA concentrations circulating in the maternal plasma between mothers delivering term and preterm neonates, across gestation using a longitudinal study design. This descriptive study identifies the miRNA content in exosomes present in maternal plasma of term and preterm birth (PTB) (n = 20 and n = 10 per each gestational period, respectively) across gestation (*i.e.*, first, second, and third trimesters and at the time of delivery). Changes in exosomal miRNA signature in maternal plasma during term and preterm gestation were determined using the NextSeq 500 high-output 75 cycles sequencing platform. A total of 167 and 153 miRNAs were found to significantly change (*P* < 0.05) as a function of the gestational age across term and PTB pregnancies, respectively. Interestingly, a comparison analysis between the exosomal miRNA profile between term and PTB reveals a total of 173 miRNAs that significantly change (*P* < 0.05) across gestation. Specific trends of changes (*i.e.*, increase, decrease, and both) as a function of the gestational age were also identified. The bioinformatics analyses establish that the differences in the miRNA profile are targeting signaling pathways associated with TGF-*β* signaling, p53, and glucocorticoid receptor signaling, respectively. These data suggest that the miRNA content of circulating exosomes in maternal blood might represent a biomolecular “fingerprint” of the progression of pregnancy.

Normal term human parturition is initiated when fetal organ systems are matured at any time between 37 and 40 weeks of gestation. Conventional theories of parturition initiation signaling are primarily linked to fetomaternal endocrine and immune changes in the intrauterine cavity, correlating with fetal growth and development (1). Homeostatic imbalances produced by these changes, specifically biochemicals released by maturing fetal organs, can lead to an inflammatory overload that disrupts the maintenance of pregnancy, resulting in labor-related changes (2–4). More precise knowledge of these signals and their mechanisms in normal term pregnancies can provide insight into pathologic activation of these signals, which can cause spontaneous preterm parturitions. Besides the fetus, fetal components of the intrauterine tissues (fetal membranes and placenta) also contribute to parturition initiation signals. Recently, we and others have reported that senescence of the placenta/membranes generates “sterile inflammation” that can enhance inflammatory load in the maternal uterine tissues capable of triggering parturition (5).

Communication between the fetal tissues and maternal organs is fundamental for a healthy pregnancy, and recently the participation of extracellular vesicles, especially in a specific type of extracellular vesicles originated from endosomal compartments, called exosomes, in cell-to-cell communication has been identified (6, 7). We have recently identified the fetal-derived signaling through exosomes by senescent fetal membrane (amniochorion) cells at term (8). Exosomes transport materials from cells and function as intercellular communication channels. Exosomes are generated by the intraluminal invagination of early endosomes, giving rise to multivesicular bodies and released into the extracellular environment upon the fusion of multivesicular bodies with the plasma membranes (9–11). Exosome contents represent the character, that is, the physiologic and metabolic state, of the cell of origin. This makes the exosomes a good vector of paracrine signaling as well as biochemical indicators of function of the cell of origin (9). Therefore, the placenta and placental membrane cells provide functional contributions during pregnancy and signal their status through exosomes, contributing to the pregnancy and promotion of fetal delivery (as well as their own) when the fetus is mature (12).

Exosomes are involved in cell-to-cell signaling mainly due to their inherent property to carry bioactive molecules (consisting of proteins, bioactive lipids, and RNAs), which are delivered to the target cells to regulate the biological functions. Interestingly, exosomes are enriched in small noncoding RNA such as miRNAs (13). miRNAs are a class of small (∼22 nucleotides long) noncoding RNAs that negatively regulate gene expression by binding to the 3′ untranslated region of target mRNAs. Interestingly, several studies have demonstrated that changes in the maternal circulating miRNAs or in placental tissues might reflect the maternal metabolic state that may be used as biomarkers of pregnancy complications. Functional studies have also shown the roles of miRNAs in regulating myometrial contractility during parturition (14). However, most studies have been conducted on identifying the total circulating miRNAs (*i.e.*, a mix of soluble, protein-associated, and vesicle-associated miRNAs) and at single time point during gestation, as a cross-sectional study design.

Multiple reports have identified the usefulness of placental-derived exosomes in predicting high-risk status for various adverse pregnancy outcomes. These include preeclampsia (15), gestational diabetes (16), and intrauterine growth restriction (17). Thus, the fetal tissue exosomes serve as proxy for underlying tissue level changes in normal and abnormal pregnancies (6). Therefore, the role of circulating exosomes in maternal blood merits further investigation. We designed a non–hypothesis-driven, descriptive study to discover exosome miRNA cargoes that are differentially expressed in total maternal plasma to generate a profile of their longitudinal changes during each stage of gestation and real-time insight into functional changes associated with gestational age in term and preterm birth (PTB) pregnancies. We also determined whether these miRNA signatures differ between preterm and term births and correlated their likely functions and reasons for their overabundance at a given state of pregnancy based on bioinformatics analysis.

## Subjects and Methods

### Study group and biospecimen collection

A hospital-based cohort of pregnant women was initiated in 2015 at a district hospital in Gurugram, Haryana (Gurugram Civil Hospital, GCH), India by the Pediatric Biology Center, Translational Health Science and Technology. Serial biospecimens are being collected across pregnancy (at the first, second, and third trimesters), at delivery, and after delivery. Ultrasound images are acquired serially during pregnancy, and the period of gestation is confirmed at enrolment by performing a dating ultrasound using standard fetal biometric parameters. Well-characterized clinical and environmental information is also maintained. The sample preparation at the study site is carried out in the research laboratory established at GCH on nationally accredited equipment from the National Accreditation Board for Testing and Calibration Laboratories. Plasma samples are stored at –80°C until analyses. All women provide written informed consent, and the study is approved by the Institute and Hospital Ethics Review Board.

### Selection of samples for this study

The selection of biospecimens for this study has been carried using a nested case control design. The cases and controls have been selected from a defined universe comprised of participants who had a normal singleton vaginal delivery, with no congenital abnormalities or associated comorbidity (*e.g.*, preeclampsia, gestational diabetes, pregnancy-induced hypertension, medical condition complicating complication) at any time in pregnancy. The cases were subjects delivered preterm defined as <37 completed weeks period of gestation. Participants who delivered at >37 completed weeks to 40 completed weeks were considered as controls. The cases and controls were matched to parity, sex of the baby, and month of delivery.

### Enrichment of exosomes from maternal blood

Exosomes were isolated from plasma as previously described (18). In brief, plasma was diluted with an equal volume of PBS (pH 7.4) and centrifuged at 2000*g* for 30 minutes at 4°C (Sorvall^®^ high-speed microcentrifuge, fixed rotor; Thermo Fisher Scientific, Asheville, NC). The 2000*g* supernatant fluid was then centrifuged at 12,000*g* for 45 minutes at 4°C (Sorvall^®^ high-speed microcentrifuge, fixed rotor; Thermo Fisher Scientific). The resultant supernatant fluid (2 mL) was transferred to an ultracentrifuge tube (10 mL; Beckman, Brea, CA) and centrifuged at 100,000*g* for 2 hours (Sorvall^®^ T-8100 fixed ultracentrifuge rotor). The 100,000*g* pellet was suspended in PBS (10 mL) and filtered through a 0.22-μm filter (Steritop™; Millipore, Billerica, MA) and then centrifuged at 100,000*g* for 2 hours. Finally, the pellet containing the enriched exosome population was resuspended in 250 μL of PBS for the characterization of exosomes and RNA extraction.

### Transmission electron microscopy

For electron microscopy analysis, exosome pellets were fixed in 3% (w/v) glutaraldehyde and analyzed under an FEI Tecnai 12 transmission electron microscope (FEI, Hillsboro, OR) as we previously described (19).

### Western blot

Western blot was performed to ascertain the identity of the exosomes using exosomal-specific markers (TSG101, CD9, and CD63) as well as Golgi marker Grp94, which is not enriched in exosomes. Standard Western blot protocols were used and the detailed protocols can be seen our prior reports (15, 20–22). An Odyssey^®^ CLx imaging system (LI-COR Biosciences, Lincoln, NE) was used to visualize the fluorescent bands.

### Nanoparticle tracking analysis

Nanoparticle tracking analyses were performed using a NanoSight NS500 instrument (NanoSight NTA 2.3 nanoparticle tracking and analysis release version build 0033; Malvern Instruments, Malvern, United Kingdom) following the manufacturer’s instructions. Samples were diluted 1:1000 with Dulbecco’s PBS to obtain 10:100 particles per image (optimal ∼50 particles per image). The NS500 instrument measured the rate of Brownian motion of nanoparticles in a light-scattering system that provides a reproducible platform for specific and general nanoparticle characterization (NanoSight, Amesbury, United Kingdom). Suspended nanoparticles were passed through the laser beam that traversed the sample chamber and scattered the light, discerning the nanoparticles through a microscope. The samples were mixed before introducing them into the chamber (temperature, 25°C), and the camera level was set to obtain an image that had sufficient contrast to clearly identify particles while minimizing background noise (camera level, 10; capture duration, 30 seconds). The captured videos (two videos per sample) were then processed and analyzed. Each video file was processed and analyzed to give the mean and mode of particle sizes, along with the concentration and the number of particles. An Excel spreadsheet was automatically generated and data were imported into GraphPad Prism 7 (La Jolla, CA).

### Exosomal RNA isolation

Exosomal RNA was extracted using an RNeasy Mini Kit 50 (Qiagen, Hilden, Germany) and TRIzol LS reagent (Life Technologies, Carlsbad, CA). The manufacturers’ protocols were followed, with slight modifications in accordance with a protocol used previously (20, 23). A spectrophotometer (SPECTROstar Nano microplate reader; BMG Labtech, Cary, NC) was used to quantify RNA concentration. Following a cleanliness check and blank measurement using RNase-free water, 2 μL of sample was pipetted onto each microdrop well on an LVis plate. RNA concentration was measured using MARS data analysis microplate reader software.

### Next-generation sequencing

Sequencing libraries were generated using the TruSeq Small RNA Library Prep Kit (Illumina, San Diego, CA), according to the manufacturer’s instructions, and as previously described (19, 20). Two hundred to 800 ng of exosomal RNA was used as input for library preparation. These RNA samples were barcoded by ligation with unique adaptor sequences to allow pooling of samples in groups of 24. Subsequently, these ligated samples were reverse transcribed, PCR amplified, and size selected using gel electrophoresis. Finally, the DNA libraries were eluted from the gel pieces overnight in 200 μL of nuclease-free H_2_O. The elution containing the pooled DNA library was further processed for cluster generation and sequencing using a NextSeq 500 high-output kit 75 cycles and Illumina NextSeq 500 sequencing platform, respectively.

### Statistical analysis and bioinformatics analysis

The number of exosomes released from individuals are represented as mean ± SE (n = 20 for term and n = 10 for PTB, independent isolations from each patient from each trimester). For RNA sequencing, first the FASTQ file generated from next-generation sequencing was preprocessed to remove adaptor sequences using the TagCleaner program (http://edwards.sdsu.edu/tagcleaner). Subsequently, known and novel miRNAs were detected within the preprocessed FASTQ data using the miRDeep2 program (24). After detection, the miRNA counts and corresponding miRNAs were extracted from the output file. Differential expression and statistical analysis of sequencing data were performed by the DESeq2 package in R. This package uses a generalized linear model to perform differential expression. Statistical analysis and significance were calculated using various tests and adjusted for multiple testing. Adjusted statistical significance was assessed as *P* < 0.001–0.05. Sequencing data have been deposited in the Gene Expression Omnibus database under no. GSE115572. The effect of pregnancy condition (*i.e.*, normal or PTB) on the size distribution (mean and mode) of the exosomes was assessed using a pairwise *post hoc* test (Tukey honest significant difference test). No significant differences (*P* > 0.05) were observed.

## Results

### Description of the participants

Table 1 describes the comparison between the demographic and the clinical characteristics of the cases and controls. No significant differences between various sociodemographic parameters were seen between cases and controls, except for body mass index (which has been adjusted in our analysis).

**Table 1.** Clinical and Demographic Characteristics of Cases and Controls

	Controls (n = 20): N (%) or Median (IQR)	Cases (n = 10): N (%) or Median (IQR)	*P* Value
Maternal variables			
Age, y	22 (20, 24)	22 (20, 23)	NS
Weight, kg	49.3 (46.8, 55.0)	39.1 (37.0, 43.2)	
Height, cm	154.0 (150.0, 158.7)	151.8 (146.6, 157.2)	NS
BMI, kg/m^2^	20.8 (19.9, 23.9)	17.6 (16.0, 19.0)	≤0.001
Period of gestation*^a^* at enrollment	12^+1^ (8^+2^, 13^+1^)	9^+2^ (7^+1^, 12^+1^)	NS
Period of gestation*^a^* at visit 2 (between 18 and 20 wk of gestation)	18^+6^ (18^+4^, 19^+0^)	18^+6^ (18^+5^, 19^+2^)	NS
Period of gestation*^a^* at visit 3 (between 26 and 28 wk of gestation)	26^+2^ (26^+2^, 26^+4^)	26^+3^ (26^+2^, 26^+5^)	NS
Period of gestation*^a^* at delivery	39^+2^ (38^+6^, 40^+2^)	36^+0^ (35^+0^, 36^+3^)	≤0.001
Newborn variables			
Placental weight, g	460.0 (405.0, 532.5)	377.5 (328.0, 433.0)	≤0.01
Baby weight, g	3013.0 (2460.5, 3265.5)	2148.0 (1980.0, 2442.0)	≤0.001

Data are presented as N (%) or median (IQR). All pregnancies were normal singleton vaginal delivery with no congenital abnormalities and with no associated comorbidity at any time in pregnancy. The cases and controls were matched to parity, sex of the baby, and month of delivery. A standard two-tailed Student *t* test was used for comparisons between control and case samples. Statistical significance was assessed as *P* < 0.001–0.05. Statistical significance was found in maternal variables (BMI, period of gestation at delivery) and newborn variables (placental weight, baby weight).

Abbreviations: BMI, body mass index; IQR, interquartile range; NS, not significant.

^a^ Period of gestation is calculated by dating ultrasound performed at the time of enrollment.

### Exosome isolation and characterization

**Figure 1.**
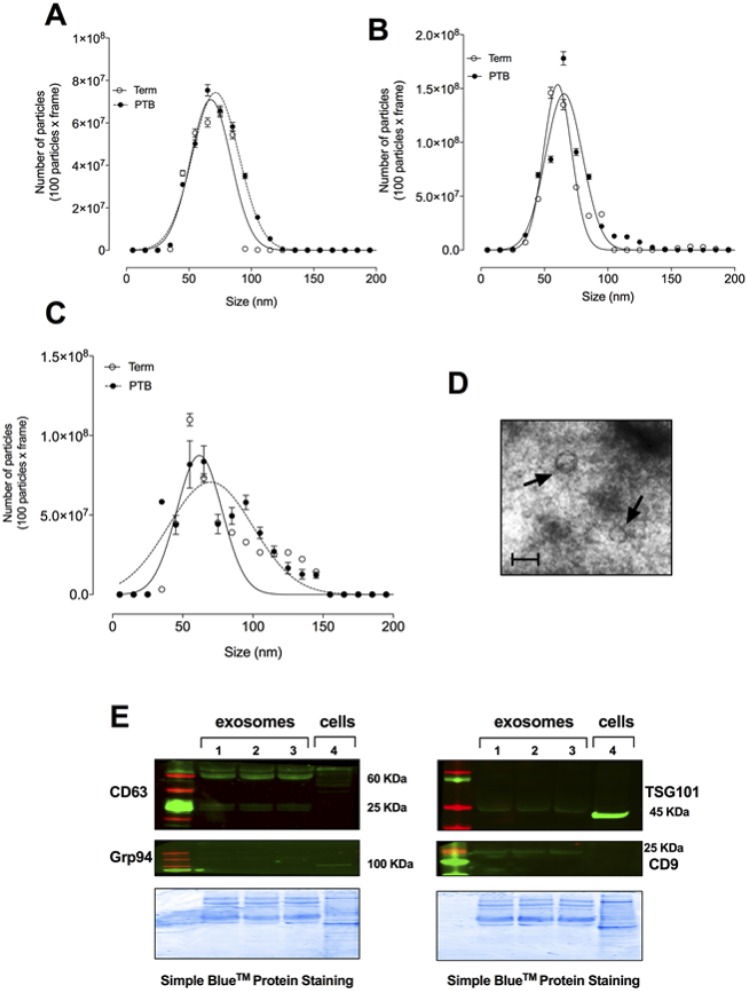
Isolation of exosomes. Exosomes were isolated from maternal plasma obtained from normal and PTB pregnancies across gestation (see “Subjects and Methods”). (A–C) Graphical representation of the vesicle size distribution using a NanoSight NS500 instrument at different time points during pregnancy [(A) first trimester, (B) second trimester, and (C) third trimester]. (D) Representative image of electron micrograph of exosomes. Scale bar, 100 nm. (E) Representative Western blot for enriched exosome markers CD63, CD9, and TSG101, as well as negative control Grp94 for exosomes isolated from normal and PTB at different time points during pregnancy. In (A)–(C) and (E), none of the experiments performed was significantly different for normal vs PTB.

Particles between 30 and 120 nm were identified on nanoparticle tracking analysis and analyzed per trimester (Fig. 1A–1C), and the cup-shaped morphology was identified using electron microscopy (Fig. 1D). Exosomes were found to be positive for CD63, CD9, and TSG101 (Fig. 1E) but negative for Golgi marker Grp94. No differences were observed in size distribution, protein abundance (CD9, CD63, and TSG101), and morphology between exosomes isolated from term and PTB.

### Exosomal miRNA signature across normal pregnancy

**Figure 2.**
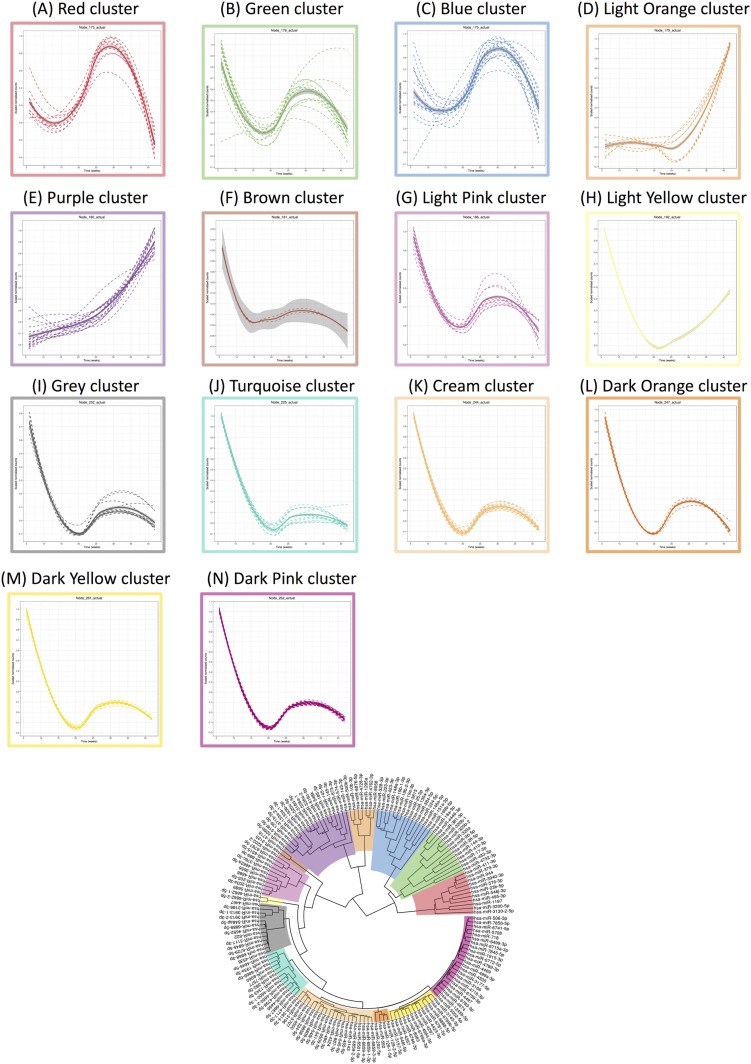
Linear mixed modeling of 167 statistically significant miRNAs that change across gestation for normal pregnancies. miRNA counts were normalized using the DESeq2 package in R prior to statistical analysis using the likelihood ratio test. Subsequently, linear mixed modeling was performed on the 167 statistically significant miRNAs (*P* < 0.05) that change across gestation for normal pregnancies, using the lme4 package in R. The data were scaled between 0 and 1 before hierarchical clustering analysis using Euclidean distance, which is displayed as a circular cladogram (generated using the ggtree package in R). Each color of the circular cladogram represents a different cluster and its trend, as shown in (A)–(N).

**Figure 3.**
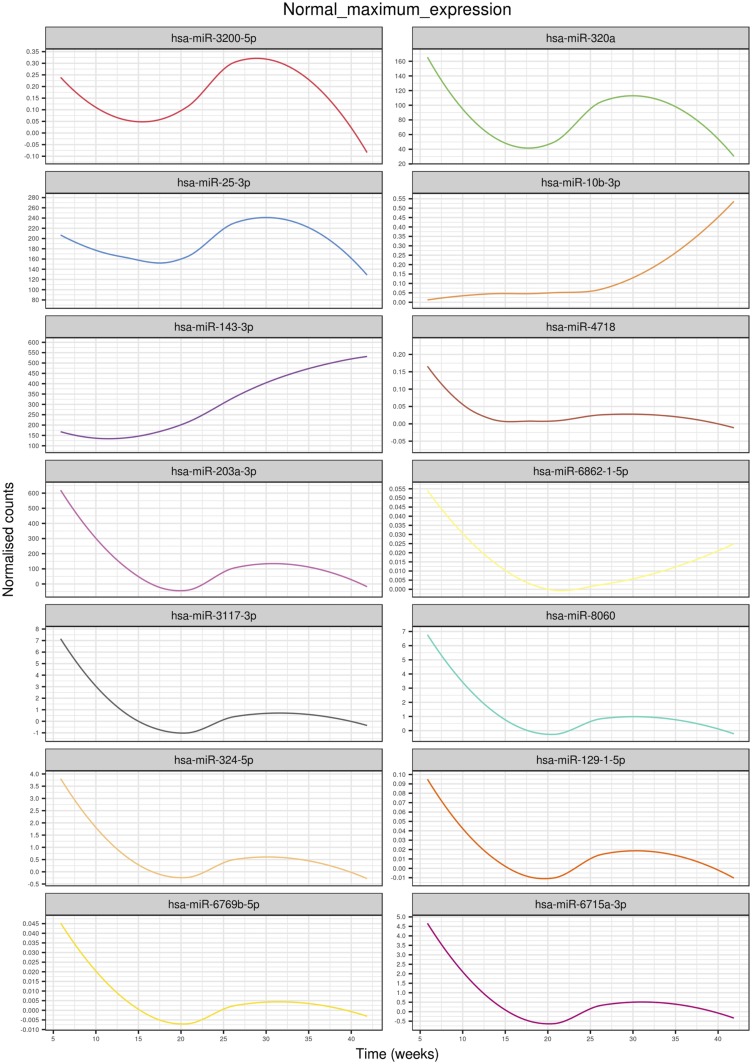
Highest expressed miRNAs in each cluster identified using linear mixed modeling for normal pregnancies across gestation. Linear mixed modeling and circular cladogram analysis for normal pregnancies across gestation were divided into 14 clusters. The highest expressed miRNA from each cluster was extracted and color coded based on the cluster of origin. Clusters are in ascending order [corresponding to (A)–(N) in Fig. 2] starting from the top of the figure, reading from left to right and continuing onto the next line of graphs below.

A total of 167 miRNAs across gestation in term pregnancies revealed significant changes (*P* < 0.05). Hierarchical clustering analysis of the average miRNA expression profiles across gestation revealed a variety of trends (Fig. 2). Specifically, trends that increased to a maximum peak expression in the third trimester are clusters A and C (A = 11, C = 15); trends that rapidly decreased in expression until the end of the first trimester and then increased to a peak at the end of the second trimester are clusters B, F, G, I, J, K, L, M, and N (B = 13, F = 2, G = 11, I = 13, J = 13, K = 21, L = 4, M = 13, N = 23); an increasing trend throughout gestation was identified in cluster E (E = 19); a trend that rapidly decreased in expression until the end of the first trimester and then increased in expression until the end of the third trimester was observed for cluster H (H = 2); and a trend that increased rapidly from the beginning of the third trimester is cluster D (D = 7). miRNAs with the largest maximum expression in each cluster are as follows (Fig. 3; Table 2): A, hsa-miR-3200-5p; B, hsa-miR-320a; C, hsa-miR-25-3p; D, hsa-miR-10b-3p; E, hsa-miR-143-3p; F, hsa-miR-4718; G, hsa-miR-203a-3p; H, hsa-miR-6862-1-5p; I, hsa-miR-3117-3p; J, hsa-miR-8060; K, hsa-miR-324-5p; L, hsa-miR-129-1-5p; M, hsa-miR-6769b-5p; and N, hsa-miR-6715a-3p.

**Table 2.** Significant miRNAs That Change Across Gestation for Normal Pregnancies

Condition	miRNA	Gestational Age With Minimum Expression	Minimum Expression (Normalized Counts)	Gestational Age With Maximum Expression	Maximum Expression (Normalized Counts)	*P* Value	Letter
Normal	hsa-miR-3200-5p	41.857	−0.034	26.143	0.321	0.036	A
Normal	hsa-miR-379-3p	41.857	−0.064	28.143	0.269	0.016	A
Normal	hsa-miR-411-3p	41.857	−0.070	27.000	0.278	0.015	A
Normal	hsa-miR-576-3p	41.857	−0.021	28.143	0.210	0.025	A
Normal	hsa-miR-1197	13.571	−0.001	28.143	0.068	0.046	A
Normal	hsa-miR-495-3p	12.143	−0.019	28.143	0.093	0.040	A
Normal	hsa-miR-3130-2-5p	41.857	−0.030	27.000	0.090	0.016	A
Normal	hsa-miR-944	41.857	−0.026	28.143	0.092	0.047	A
Normal	hsa-miR-548j-3p	13.714	−0.009	27.000	0.062	0.017	A
Normal	hsa-miR-3940-3p	41.857	−0.024	28.143	0.071	0.037	A
Normal	hsa-miR-23b-5p	13.714	−0.003	27.000	0.012	0.016	A
Normal	hsa-miR-320a	41.857	29.996	5.857	163.935	0.026	B
Normal	hsa-miR-320b-2	41.857	21.416	5.857	145.869	0.030	B
Normal	hsa-miR-320b-1	41.857	22.168	6.143	139.071	0.030	B
Normal	hsa-miR-144-3p	40.857	−1.551	6.143	60.607	0.045	B
Normal	hsa-miR-4732-3p	19.000	1.474	6.143	13.023	0.007	B
Normal	hsa-miR-410-3p	19.000	0.419	28.143	11.632	0.007	B
Normal	hsa-miR-28-5p	40.857	1.067	6.143	10.960	0.044	B
Normal	hsa-miR-4446-3p	19.143	1.069	5.857	7.583	0.044	B
Normal	hsa-miR-96-5p	41.857	−0.245	6.143	8.217	0.025	B
Normal	hsa-miR-381-3p	19.000	0.222	6.143	7.765	0.021	B
Normal	hsa-miR-17-3p	39.571	−0.028	12.000	1.535	0.035	B
Normal	hsa-miR-18a-3p	18.714	0.116	5.857	1.629	0.023	B
Normal	hsa-miR-433-3p	18.714	0.048	39.000	0.372	0.034	B
Normal	hsa-miR-25-3p	39.571	39.144	38.857	342.593	0.039	C
Normal	hsa-miR-151a-3p	40.857	53.696	27.000	293.888	0.044	C
Normal	hsa-miR-148a-3p	13.857	72.308	27.000	321.138	0.005	C
Normal	hsa-miR-146a-5p	41.857	33.933	27.429	206.489	0.016	C
Normal	hsa-miR-423-3p	13.571	22.310	27.429	128.235	0.011	C
Normal	hsa-miR-223-3p	13.857	8.677	27.000	89.880	0.011	C
Normal	hsa-miR-584-5p	13.857	13.667	27.000	63.942	0.007	C
Normal	hsa-miR-19b-2-3p	13.857	3.510	28.143	22.076	0.028	C
Normal	hsa-miR-19b-1-3p	13.857	3.649	28.143	22.046	0.028	C
Normal	hsa-miR-19a-3p	13.857	4.293	28.143	21.087	0.036	C
Normal	hsa-miR-3615	13.571	2.770	37.000	9.088	0.038	C
Normal	hsa-miR-328-3p	11.286	0.815	37.000	5.739	0.006	C
Normal	hsa-miR-1304-3p	12.429	0.631	27.000	1.946	0.036	C
Normal	hsa-miR-224-5p	19.000	0.412	27.000	2.165	0.027	C
Normal	hsa-miR-199b-5p	6.143	−0.083	27.429	0.977	0.032	C
Normal	hsa-miR-10b-3p	6.429	−0.002	41.857	0.513	0.041	D
Normal	hsa-miR-500b-3p	20.571	0.001	41.857	0.066	0.012	D
Normal	hsa-miR-4728-3p	19.000	−0.004	41.857	0.045	0.027	D
Normal	hsa-miR-6876-5p	12.143	−0.004	41.857	0.043	0.038	D
Normal	hsa-miR-8058	26.286	−0.005	41.857	0.028	0.021	D
Normal	hsa-miR-4762-3p	26.571	−0.003	41.857	0.015	0.021	D
Normal	hsa-miR-1295a	27.429	−0.005	41.857	0.029	0.021	D
Normal	hsa-miR-143-3p	19.000	53.724	37.000	611.513	0.001	E
Normal	hsa-miR-133a-1-3p	12.571	−0.252	40.429	7.449	0.000	E
Normal	hsa-miR-133a-2-3p	10.286	−0.429	40.429	7.416	0.000	E
Normal	hsa-miR-574-3p	19.000	0.551	40.429	3.305	0.043	E
Normal	hsa-miR-145-3p	8.571	0.347	41.857	2.804	0.002	E
Normal	hsa-miR-145-5p	6.143	0.107	41.857	3.038	0.000	E
Normal	hsa-miR-518d-5p	6.286	−0.162	39.000	2.442	0.041	E
Normal	hsa-miR-526a-1	5.857	−0.016	39.000	2.255	0.041	E
Normal	hsa-miR-526a-2	6.286	−0.069	39.000	2.443	0.041	E
Normal	hsa-miR-520c-5p	6.429	−0.144	39.000	2.291	0.040	E
Normal	hsa-miR-185-3p	6.143	0.156	41.857	2.075	0.048	E
Normal	hsa-miR-371b-5p	6.286	0.034	41.857	1.504	0.005	E
Normal	hsa-miR-548k	6.286	0.009	39.000	1.300	0.047	E
Normal	hsa-miR-518f-5p	6.429	−0.117	41.857	1.693	0.006	E
Normal	hsa-miR-526b-5p	5.857	−0.026	41.857	1.468	0.022	E
Normal	hsa-miR-518c-5p	6.286	−0.097	39.000	1.278	0.040	E
Normal	hsa-miR-193b-3p	6.143	−0.004	40.429	0.860	0.017	E
Normal	hsa-miR-873-3p	20.571	0.035	41.857	0.338	0.024	E
Normal	hsa-miR-139-3p	10.286	0.001	41.857	0.273	0.047	E
Normal	hsa-miR-4718	20.571	−0.025	5.857	0.470	0.045	F
Normal	hsa-miR-3125	18.714	−0.016	5.857	0.250	0.008	F
Normal	hsa-miR-203a-3p	20.286	−53.378	5.857	609.717	0.038	G
Normal	hsa-miR-205-5p	18.571	−1.562	6.286	18.783	0.039	G
Normal	hsa-miR-5689	19.143	−0.980	6.143	13.261	0.035	G
Normal	hsa-miR-6767-5p	41.857	−0.933	6.143	7.801	0.033	G
Normal	hsa-miR-889-3p	20.286	−0.202	6.143	6.763	0.009	G
Normal	hsa-miR-4662a-5p	41.857	−0.301	5.857	2.447	0.028	G
Normal	hsa-miR-376c-3p	20.286	0.000	5.857	0.309	0.017	G
Normal	hsa-miR-3656	41.857	−0.025	5.857	0.188	0.014	G
Normal	hsa-miR-6515-5p	41.857	−0.017	5.857	0.129	0.029	G
Normal	hsa-miR-4688	18.714	−0.008	5.857	0.063	0.039	G
Normal	hsa-miR-3202-2	41.857	−0.005	6.286	0.023	0.048	G
Normal	hsa-miR-6862-1-5p	20.571	−0.003	5.857	0.054	0.031	H
Normal	hsa-miR-6862-2-5p	20.571	−0.004	5.857	0.055	0.031	H
Normal	hsa-miR-3117-3p	19.143	−1.534	6.143	9.596	0.018	I
Normal	hsa-miR-6845-5p	20.286	−0.656	6.143	4.733	0.020	I
Normal	hsa-miR-6889-5p	20.286	−0.226	6.143	1.603	0.021	I
Normal	hsa-miR-548ap-5p	20.286	−0.245	6.143	1.582	0.025	I
Normal	hsa-miR-6868-3p	19.000	−0.256	6.143	1.717	0.022	I
Normal	hsa-miR-6729-5p	18.857	−0.215	6.143	1.716	0.014	I
Normal	hsa-miR-622	20.286	−0.239	6.143	1.735	0.022	I
Normal	hsa-miR-4538	20.571	−0.215	6.143	1.610	0.022	I
Normal	hsa-miR-4652-5p	20.286	−0.224	6.143	1.632	0.022	I
Normal	hsa-miR-3913-1-3p	20.286	−0.010	5.857	0.060	0.037	I
Normal	hsa-miR-3913-2-3p	18.714	−0.008	5.857	0.059	0.037	I
Normal	hsa-miR-4467	20.286	−0.011	5.857	0.096	0.004	I
Normal	hsa-miR-3186-3p	19.143	−0.032	5.857	0.199	0.021	I
Normal	hsa-miR-8060	18.571	−0.290	6.143	6.667	0.049	J
Normal	hsa-miR-7977	41.857	−0.204	5.857	5.111	0.037	J
Normal	hsa-miR-193a-3p	41.857	−0.092	6.286	1.340	0.026	J
Normal	hsa-miR-6869-5p	41.857	−0.126	6.143	1.292	0.026	J
Normal	hsa-miR-4798-5p	19.000	−0.125	5.857	1.247	0.027	J
Normal	hsa-miR-4466	18.429	−0.105	6.429	1.140	0.032	J
Normal	hsa-miR-4646-5p	20.286	−0.067	6.143	1.140	0.047	J
Normal	hsa-miR-382-5p	19.000	−0.031	5.857	0.352	0.005	J
Normal	hsa-miR-6867-5p	20.286	−0.003	6.143	0.018	0.030	J
Normal	hsa-miR-4650-1-3p	41.857	−0.002	6.286	0.024	0.013	J
Normal	hsa-miR-4650-2-3p	19.143	−0.003	6.143	0.024	0.013	J
Normal	hsa-miR-1343-3p	18.857	−0.001	5.857	0.008	0.040	J
Normal	hsa-miR-4717-3p	19.000	−0.002	5.857	0.013	0.019	J
Normal	hsa-miR-324-5p	19.143	−0.289	5.857	3.734	0.022	K
Normal	hsa-miR-5707	19.000	−0.487	5.857	3.490	0.020	K
Normal	hsa-miR-3614-3p	19.000	−0.738	5.857	4.524	0.026	K
Normal	hsa-miR-3196	18.714	−0.341	5.857	2.480	0.018	K
Normal	hsa-miR-6509-5p	20.286	−0.306	5.857	2.429	0.026	K
Normal	hsa-miR-4324	19.143	−0.595	5.857	3.828	0.015	K
Normal	hsa-miR-455-5p	19.000	−0.437	6.143	3.332	0.032	K
Normal	hsa-miR-4500	41.857	−0.122	6.286	1.190	0.029	K
Normal	hsa-miR-6501-5p	18.714	−0.342	5.857	2.237	0.025	K
Normal	hsa-miR-887-3p	20.571	−0.322	5.857	2.344	0.023	K
Normal	hsa-miR-3143	18.429	−0.161	5.857	1.232	0.014	K
Normal	hsa-miR-548j-5p	19.143	−0.135	5.857	1.107	0.049	K
Normal	hsa-miR-490-3p	19.143	−0.132	5.857	1.145	0.033	K
Normal	hsa-miR-6858-5p	41.857	−0.019	6.143	0.241	0.027	K
Normal	hsa-miR-545-5p	20.286	−0.021	5.857	0.186	0.000	K
Normal	hsa-miR-6809-5p	20.571	−0.004	6.143	0.058	0.019	K
Normal	hsa-miR-541-3p	19.000	−0.010	5.857	0.095	0.010	K
Normal	hsa-miR-6859-1-3p	20.286	−0.009	5.857	0.053	0.003	K
Normal	hsa-miR-6859-2-3p	18.429	−0.006	5.857	0.052	0.003	K
Normal	hsa-miR-6859-3-3p	18.857	−0.007	5.857	0.055	0.003	K
Normal	hsa-miR-6859-4-3p	20.286	−0.009	5.857	0.055	0.003	K
Normal	hsa-miR-129-1-5p	20.571	−0.013	5.857	0.097	0.021	L
Normal	hsa-miR-129-2-5p	19.000	−0.014	5.857	0.103	0.021	L
Normal	hsa-miR-202-5p	20.286	−0.016	5.857	0.125	0.003	L
Normal	hsa-miR-592	40.857	−0.005	5.857	0.048	0.038	L
Normal	hsa-miR-6769b-5p	18.571	−0.008	5.857	0.046	0.015	M
Normal	hsa-miR-548t-5p	20.571	−0.007	5.857	0.045	0.025	M
Normal	hsa-miR-4297	20.571	−0.017	5.857	0.090	0.015	M
Normal	hsa-miR-6781-5p	20.571	−0.001	6.143	0.005	0.026	M
Normal	hsa-miR-6888-5p	19.000	0.000	5.857	0.002	0.026	M
Normal	hsa-miR-4684-3p	19.000	−0.005	6.143	0.036	0.031	M
Normal	hsa-miR-8083	18.857	−0.003	5.857	0.018	0.031	M
Normal	hsa-miR-3145-3p	18.571	0.000	5.857	0.002	0.026	M
Normal	hsa-miR-4267	18.714	−0.002	6.143	0.017	0.031	M
Normal	hsa-miR-6769a-3p	18.714	−0.003	5.857	0.018	0.031	M
Normal	hsa-miR-4423-3p	19.000	0.000	6.143	0.002	0.026	M
Normal	hsa-miR-6766-3p	18.857	−0.001	5.857	0.005	0.026	M
Normal	hsa-miR-3157-5p	18.429	0.000	5.857	0.002	0.026	M
Normal	hsa-miR-6715a-3p	19.000	−0.795	5.857	4.636	0.023	N
Normal	hsa-miR-1915-3p	20.286	−0.594	5.857	3.537	0.024	N
Normal	hsa-miR-4469	20.286	−0.016	6.143	0.085	0.013	N
Normal	hsa-miR-4535	18.857	−0.008	5.857	0.052	0.020	N
Normal	hsa-miR-7856-5p	19.143	−0.006	6.286	0.043	0.019	N
Normal	hsa-miR-7845-5p	19.000	−0.017	5.857	0.087	0.015	N
Normal	hsa-miR-4789-3p	19.000	−0.009	5.857	0.043	0.015	N
Normal	hsa-miR-6772-5p	20.286	0.000	5.857	0.002	0.026	N
Normal	hsa-miR-4716-3p	18.714	0.000	5.857	0.002	0.026	N
Normal	hsa-miR-6783-3p	19.000	0.000	6.143	0.002	0.026	N
Normal	hsa-miR-499a-3p	18.714	−0.012	6.143	0.067	0.031	N
Normal	hsa-miR-718	19.000	−0.007	5.857	0.049	0.013	N
Normal	hsa-miR-3177-5p	19.143	−0.001	6.429	0.002	0.026	N
Normal	hsa-miR-5002-5p	19.143	−0.003	5.857	0.017	0.031	N
Normal	hsa-miR-3166	18.857	−0.006	5.857	0.034	0.031	N
Normal	hsa-miR-6499-3p	19.143	−0.003	5.857	0.017	0.031	N
Normal	hsa-miR-4674	19.000	−0.003	5.857	0.016	0.031	N
Normal	hsa-miR-4497	19.143	−0.003	5.857	0.017	0.031	N
Normal	hsa-miR-5708	19.143	−0.002	5.857	0.017	0.031	N
Normal	hsa-miR-6759-3p	18.714	−0.003	5.857	0.017	0.031	N
Normal	hsa-miR-506-3p	20.286	−0.001	6.286	0.005	0.026	N
Normal	hsa-miR-6741-5p	18.714	−0.001	5.857	0.005	0.026	N
Normal	hsa-miR-1245b-5p	20.286	0.000	6.143	0.002	0.026	N

### Exosomal miRNA signature across PTB pregnancy

**Figure 4.**
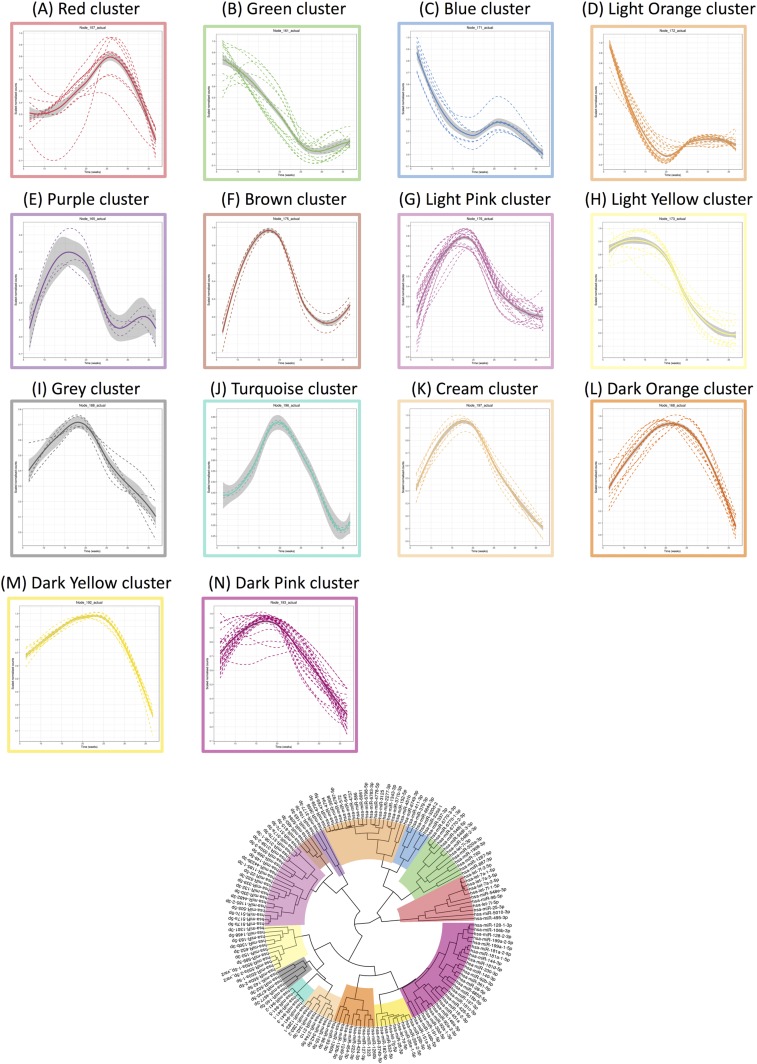
Linear mixed modeling of 153 statistically significant miRNAs that change across gestation for PTB pregnancies. miRNA counts were normalized using the DESeq2 package in R prior to statistical analysis using the likelihood ratio test. Subsequently, linear mixed modeling was performed on the 153 statistically significant miRNAs (*P* < 0.05) that change across gestation for PTB pregnancies, using the lme4 package in R. The data were scaled between 0 and 1 before hierarchical clustering analysis using Euclidean distance, which is displayed as a circular cladogram (generated using the ggtree package in R). Each color of the circular cladogram represents a different cluster and its trend, as shown in (A)–(N).

**Figure 5.**
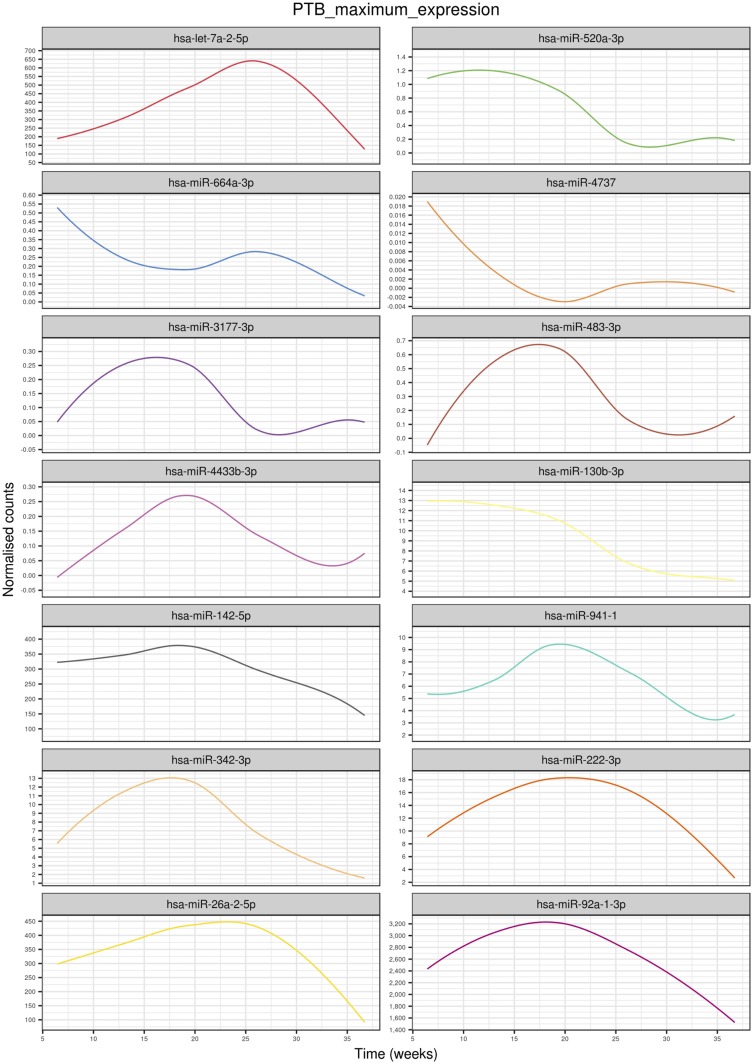
Highest expressed miRNAs in each cluster identified using linear mixed modeling for PTB pregnancies across gestation. Linear mixed modeling and circular cladogram analysis for normal pregnancies across gestation were divided into 14 clusters. The highest expressed miRNA from each cluster was extracted and color coded based on the cluster of origin. Clusters are in ascending order [corresponding to (A)–(N) in Fig. 4] starting from the top of the figure, reading from left to right and continuing onto the next line of graphs below.

A total of 153 miRNAs across gestation in PTB pregnancies demonstrated significant changes (*P* < 0.05). Hierarchical clustering analysis of the average miRNA expression profiles across gestation revealed a variety of trends (Fig. 4). Specifically, trends that had peak expression at the second trimester are clusters E, F, G, I, J, K, L, M, and N (E = 3, F = 4, G = 21, I = 5, J = 5, K = 8, L = 10, M = 9, N = 28); a trend that had peak expression at the beginning of the third trimester was identified in cluster A (A = 11); and decreasing trends in expression across gestation were observed for clusters B, C, D, and H (B = 12, C = 6, D = 20, H = 11). miRNAs with the largest maximum expression in each cluster are as follows (Fig. 5; Table 3): A, hsa-let-7a-2-5p; B, hsa-miR-520a-3p; C, hsa-miR-664a-3p; D, hsa-miR-4737; E, hsa-miR-3177-3p; F, hsa-miR-483-3p; G, hsa-miR-4433b-3p; H, hsa-miR-130b-3p; I, hsa-miR-142-5p; J, hsa-miR-941-1; K, hsa-miR-342-3p; L, hsa-miR-222-3p; M, hsa-miR-26a-2-5p; and N, hsa-miR-92a-1-3p.

**Table 3.** Significant miRNAs That Change Across Gestation for PTB Pregnancies

Condition	miRNA	Gestational Age With Minimum Expression	Minimum Expression (Normalized Counts)	Gestational Age With Maximum Expression	Maximum Expression (Normalized Counts)	*P* Value	Letter
PTB	hsa-let-7a-2-5p	36.714	83.371	26.857	765.758	0.021	A
PTB	hsa-let-7a-1-5p	36.000	112.699	26.857	783.444	0.020	A
PTB	hsa-let-7a-3-5p	36.571	111.885	26.857	779.513	0.020	A
PTB	hsa-let-7f-2-5p	36.714	87.346	26.857	756.329	0.020	A
PTB	hsa-let-7f-1-5p	36.571	108.247	26.857	772.266	0.020	A
PTB	hsa-miR-25-3p	36.714	−14.331	7.857	345.282	0.002	A
PTB	hsa-let-7i-5p	36.000	−4.748	26.571	251.392	0.019	A
PTB	hsa-miR-98-5p	36.571	0.600	26.857	18.092	0.031	A
PTB	hsa-miR-548e-3p	36.714	−0.045	26.714	0.333	0.009	A
PTB	hsa-miR-5010-3p	35.000	−0.079	19.429	0.423	0.028	A
PTB	hsa-miR-495-3p	12.143	−0.008	26.714	0.041	0.044	A
PTB	hsa-miR-520a-3p	26.857	−0.155	7.857	1.682	0.002	B
PTB	hsa-let-7i-3p	26.714	−0.044	7.857	0.871	0.007	B
PTB	hsa-miR-887-3p	32.571	−0.019	13.000	0.144	0.024	B
PTB	hsa-miR-1287-5p	35.000	−0.039	9.714	0.317	0.005	B
PTB	hsa-miR-548f-2-3p	26.714	−0.009	7.857	0.227	0.027	B
PTB	hsa-miR-548f-3-3p	26.714	−0.010	7.857	0.240	0.027	B
PTB	hsa-miR-760	32.571	0.010	7.143	0.434	0.005	B
PTB	hsa-miR-548j-5p	26.857	−0.007	6.429	0.119	0.038	B
PTB	hsa-miR-1908-3p	32.571	0.028	6.571	0.295	0.020	B
PTB	hsa-miR-6770-1-3p	26.286	−0.005	6.429	0.054	0.031	B
PTB	hsa-miR-6770-2-3p	26.857	−0.004	6.429	0.056	0.031	B
PTB	hsa-miR-6770-3-3p	26.286	−0.005	6.571	0.055	0.031	B
PTB	hsa-miR-664a-3p	36.714	−0.033	6.571	0.570	0.012	C
PTB	hsa-miR-320d-1	36.714	−0.085	6.571	0.859	0.026	C
PTB	hsa-miR-320d-2	36.714	−0.108	6.571	0.849	0.026	C
PTB	hsa-miR-379-3p	36.714	−0.053	6.571	0.599	0.036	C
PTB	hsa-miR-411-3p	36.571	−0.052	6.571	0.636	0.038	C
PTB	hsa-miR-337-3p	36.714	−0.026	6.571	0.306	0.021	C
PTB	hsa-miR-4737	19.429	−0.003	6.429	0.020	0.042	D
PTB	hsa-miR-4778-5p	18.571	−0.019	6.571	0.240	0.020	D
PTB	hsa-miR-6749-3p	19.143	−0.009	6.429	0.081	0.030	D
PTB	hsa-miR-2277-3p	19.429	−0.035	6.571	0.246	0.017	D
PTB	hsa-miR-4794	19.143	−0.004	6.429	0.020	0.042	D
PTB	hsa-miR-3125	19.429	−0.005	6.429	0.077	0.008	D
PTB	hsa-miR-8061	19.143	−0.010	6.429	0.055	0.042	D
PTB	hsa-miR-152-5p	26.143	−0.002	6.429	0.068	0.032	D
PTB	hsa-miR-451b	26.571	−0.004	6.429	0.044	0.032	D
PTB	hsa-miR-371b-3p	35.000	−0.001	6.429	0.034	0.045	D
PTB	hsa-miR-6783-3p	18.571	−0.010	6.429	0.082	0.042	D
PTB	hsa-miR-1343-3p	32.571	−0.002	6.429	0.032	0.023	D
PTB	hsa-miR-3908	19.429	−0.007	6.429	0.038	0.042	D
PTB	hsa-miR-586	19.286	−0.008	6.571	0.038	0.042	D
PTB	hsa-miR-645	19.429	−0.003	6.429	0.019	0.042	D
PTB	hsa-miR-4769-5p	18.714	−0.004	6.429	0.019	0.042	D
PTB	hsa-miR-572	19.429	−0.003	6.429	0.019	0.042	D
PTB	hsa-miR-6759-5p	19.429	−0.004	6.571	0.019	0.042	D
PTB	hsa-miR-6797-5p	19.286	−0.004	6.571	0.020	0.042	D
PTB	hsa-miR-6798-5p	18.714	−0.004	6.429	0.019	0.042	D
PTB	hsa-miR-3177-3p	26.857	−0.045	13.000	0.323	0.029	E
PTB	hsa-miR-10a-3p	26.857	−0.046	7.857	0.390	0.046	E
PTB	hsa-miR-3656	32.571	−0.037	13.000	0.284	0.031	E
PTB	hsa-miR-483-3p	6.429	−0.037	18.571	0.668	0.014	F
PTB	hsa-miR-183-3p	6.571	−0.049	18.571	0.134	0.017	F
PTB	hsa-miR-615-3p	6.429	−0.019	18.571	0.138	0.016	F
PTB	hsa-miR-5094	32.571	−0.011	18.571	0.055	0.028	F
PTB	hsa-miR-4433b-3p	6.429	−0.038	19.429	0.334	0.032	G
PTB	hsa-miR-132-3p	36.429	0.074	18.714	0.789	0.037	G
PTB	hsa-miR-3158-1-3p	36.429	−0.103	19.429	1.200	0.000	G
PTB	hsa-miR-3158-2-3p	36.429	−0.074	19.429	1.182	0.000	G
PTB	hsa-miR-339-5p	36.714	0.062	18.571	0.715	0.040	G
PTB	hsa-miR-517a-5p	36.286	−0.030	19.286	0.836	0.005	G
PTB	hsa-miR-517b-5p	36.286	0.015	19.286	0.827	0.005	G
PTB	hsa-miR-517c-5p	36.000	−0.017	19.286	0.816	0.005	G
PTB	hsa-miR-517a-3p	36.000	−0.006	18.714	0.554	0.022	G
PTB	hsa-miR-517b-3p	36.000	0.004	18.571	0.546	0.022	G
PTB	hsa-miR-517c-3p	32.571	−0.006	19.286	0.538	0.023	G
PTB	hsa-miR-532-3p	6.571	−0.002	18.571	0.397	0.046	G
PTB	hsa-miR-33b-5p	34.429	0.059	18.714	0.496	0.040	G
PTB	hsa-miR-550a-3-5p	36.000	−0.011	19.286	0.352	0.021	G
PTB	hsa-miR-369-5p	36.000	0.030	12.857	0.472	0.018	G
PTB	hsa-miR-22-5p	6.429	−0.009	18.714	0.361	0.035	G
PTB	hsa-miR-149-5p	36.000	−0.003	18.714	0.091	0.028	G
PTB	hsa-miR-4482-3p	6.429	−0.044	18.571	0.168	0.038	G
PTB	hsa-miR-1185-1-3p	6.571	−0.023	19.429	0.115	0.038	G
PTB	hsa-miR-1185-2-3p	6.571	−0.014	18.571	0.082	0.017	G
PTB	hsa-miR-505-5p	32.571	−0.006	18.714	0.125	0.028	G
PTB	hsa-miR-130b-3p	36.000	3.525	7.857	14.908	0.024	H
PTB	hsa-miR-183-5p	36.571	2.118	6.429	10.095	0.001	H
PTB	hsa-miR-652-3p	36.714	1.544	8.714	6.527	0.007	H
PTB	hsa-miR-150-3p	36.429	0.086	12.143	2.111	0.002	H
PTB	hsa-miR-1468-5p	36.000	0.105	18.571	0.900	0.027	H
PTB	hsa-miR-1301-3p	36.714	0.077	6.571	0.485	0.028	H
PTB	hsa-miR-550a-1-5p	36.571	0.017	13.000	0.489	0.027	H
PTB	hsa-miR-550a-1-5p_var2	34.429	0.037	12.857	0.486	0.027	H
PTB	hsa-miR-550a-2-5p	36.429	0.024	12.857	0.487	0.027	H
PTB	hsa-miR-550a-2-5p_var2	36.000	0.046	13.000	0.471	0.027	H
PTB	hsa-miR-589-3p	35.000	0.022	12.857	0.251	0.047	H
PTB	hsa-miR-142-5p	36.000	57.040	7.857	480.207	0.002	I
PTB	hsa-miR-502-3p	36.000	0.298	18.571	7.028	0.005	I
PTB	hsa-miR-4677-3p	36.571	−0.054	13.000	0.463	0.003	I
PTB	hsa-miR-140-5p	36.000	−0.047	13.000	0.477	0.032	I
PTB	hsa-miR-6735-5p	36.714	−0.025	18.571	0.202	0.043	I
PTB	hsa-miR-941-1	36.286	1.174	19.143	11.889	0.043	J
PTB	hsa-miR-941-2	36.286	0.880	19.143	11.888	0.043	J
PTB	hsa-miR-941-3	36.286	0.948	19.143	11.776	0.043	J
PTB	hsa-miR-941-4	36.286	1.520	19.143	11.919	0.043	J
PTB	hsa-miR-941-5	36.286	1.512	19.143	11.840	0.043	J
PTB	hsa-miR-342-3p	36.000	0.844	18.571	13.061	0.000	K
PTB	hsa-miR-155-5p	36.000	0.422	18.571	3.835	0.001	K
PTB	hsa-miR-342-5p	36.714	0.249	18.571	1.759	0.015	K
PTB	hsa-miR-374a-5p	36.714	0.078	18.571	1.270	0.004	K
PTB	hsa-miR-1283-1	36.714	0.016	19.429	0.651	0.016	K
PTB	hsa-miR-1283-2	36.714	−0.003	18.714	0.670	0.016	K
PTB	hsa-miR-98-3p	36.714	0.096	18.714	1.058	0.009	K
PTB	hsa-miR-2110	36.571	0.036	18.571	0.316	0.013	K
PTB	hsa-miR-222-3p	36.714	2.400	19.429	18.711	0.020	L
PTB	hsa-miR-1323	36.714	0.224	18.571	5.262	0.017	L
PTB	hsa-miR-424-3p	36.714	0.633	18.714	3.845	0.003	L
PTB	hsa-miR-1260b	36.571	0.114	18.571	4.723	0.003	L
PTB	hsa-miR-1260a	36.000	−0.017	19.143	3.090	0.008	L
PTB	hsa-miR-127-3p	36.714	−0.125	19.429	2.990	0.040	L
PTB	hsa-miR-454-3p	36.000	0.125	19.143	1.584	0.035	L
PTB	hsa-miR-130b-5p	36.714	0.003	26.143	1.323	0.028	L
PTB	hsa-miR-93-3p	36.714	0.098	19.286	0.701	0.018	L
PTB	hsa-miR-1249-3p	36.714	−0.003	26.857	0.378	0.013	L
PTB	hsa-miR-26a-2-5p	36.714	87.226	19.429	443.127	0.002	M
PTB	hsa-miR-26a-1-5p	36.571	106.815	19.429	448.290	0.002	M
PTB	hsa-miR-182-5p	36.571	34.092	19.143	195.739	0.001	M
PTB	hsa-miR-28-3p	36.571	28.378	19.429	127.465	0.010	M
PTB	hsa-miR-26b-5p	36.571	17.921	26.857	80.645	0.011	M
PTB	hsa-let-7g-5p	36.714	18.677	19.143	72.495	0.042	M
PTB	hsa-let-7d-5p	36.571	4.335	19.429	17.103	0.041	M
PTB	hsa-miR-532-5p	36.714	2.662	19.143	12.236	0.018	M
PTB	hsa-miR-374b-5p	36.714	0.106	19.429	1.252	0.037	M
PTB	hsa-miR-92a-1-3p	36.571	1463.034	19.143	3264.338	0.013	N
PTB	hsa-miR-92a-2-3p	36.714	1452.078	19.429	3235.394	0.016	N
PTB	hsa-miR-191-5p	36.000	313.059	18.571	1059.484	0.017	N
PTB	hsa-miR-451a	36.000	370.301	9.714	878.724	0.033	N
PTB	hsa-miR-30d-5p	36.714	129.580	12.857	285.232	0.040	N
PTB	hsa-miR-181a-1-5p	36.714	63.476	19.143	294.151	0.004	N
PTB	hsa-miR-181a-2-5p	36.571	75.583	19.429	294.918	0.004	N
PTB	hsa-miR-151a-3p	36.000	54.679	26.857	321.801	0.004	N
PTB	hsa-miR-146a-5p	36.571	59.045	7.143	195.970	0.033	N
PTB	hsa-miR-92b-3p	36.429	42.148	19.143	137.752	0.024	N
PTB	hsa-miR-146b-5p	36.000	2.335	9.714	62.211	0.017	N
PTB	hsa-miR-144-3p	36.571	9.064	18.714	35.849	0.012	N
PTB	hsa-miR-140-3p	36.286	14.249	19.143	40.063	0.048	N
PTB	hsa-miR-425-5p	36.714	9.769	12.857	38.067	0.004	N
PTB	hsa-miR-16-2-3p	36.571	5.511	13.000	18.210	0.012	N
PTB	hsa-miR-106b-3p	36.429	3.341	19.143	18.945	0.003	N
PTB	hsa-miR-128-1-3p	36.714	2.834	19.143	13.143	0.016	N
PTB	hsa-miR-128-2-3p	36.571	2.517	18.571	11.922	0.021	N
PTB	hsa-miR-181d-5p	36.429	1.657	18.571	10.690	0.009	N
PTB	hsa-miR-181c-5p	36.571	1.259	10.571	5.873	0.005	N
PTB	hsa-miR-197-3p	36.714	1.127	18.571	7.338	0.001	N
PTB	hsa-miR-15b-5p	36.000	1.635	18.571	5.638	0.034	N
PTB	hsa-miR-28-5p	34.429	2.210	18.714	5.857	0.048	N
PTB	hsa-miR-199a-1-5p	36.714	0.553	19.429	4.492	0.041	N
PTB	hsa-miR-199a-2-5p	36.571	0.864	19.143	4.406	0.041	N
PTB	hsa-miR-361-5p	36.571	1.020	18.571	3.152	0.027	N
PTB	hsa-miR-6852-5p	36.571	0.720	18.571	3.103	0.013	N
PTB	hsa-miR-330-3p	36.714	0.223	18.714	1.357	0.038	N

### Differential expression of exosomal miRNA across gestation in normal compared with PTB pregnancy

**Figure 6.**
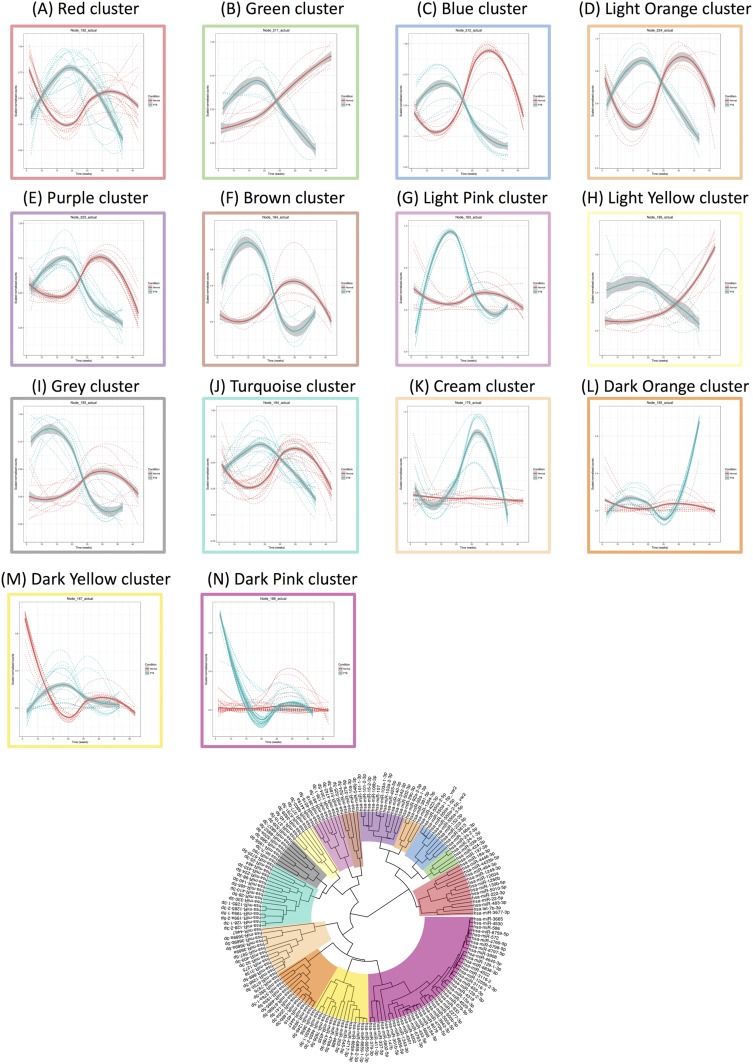
Linear mixed modeling of 173 statistically significant miRNAs that change across gestation when comparing normal to PTB pregnancies. miRNA counts were normalized using the DESeq2 package in R prior to statistical analysis using the likelihood ratio test. Subsequently, linear mixed modeling was performed on the 173 statistically significant miRNAs (*P* < 0.05) that change across gestation when comparing normal to PTB pregnancies, using the lme4 package in R. The data were scaled between 0 and 1 before hierarchical clustering analysis using Euclidean distance, which is displayed as a circular cladogram (generated using the ggtree package in R). Each color of the circular cladogram represents a different cluster and its trend, as shown in (A)–(N). Within the panels, red indicates normal pregnancies whereas blue indicates PTB pregnancies.

**Figure 7.**
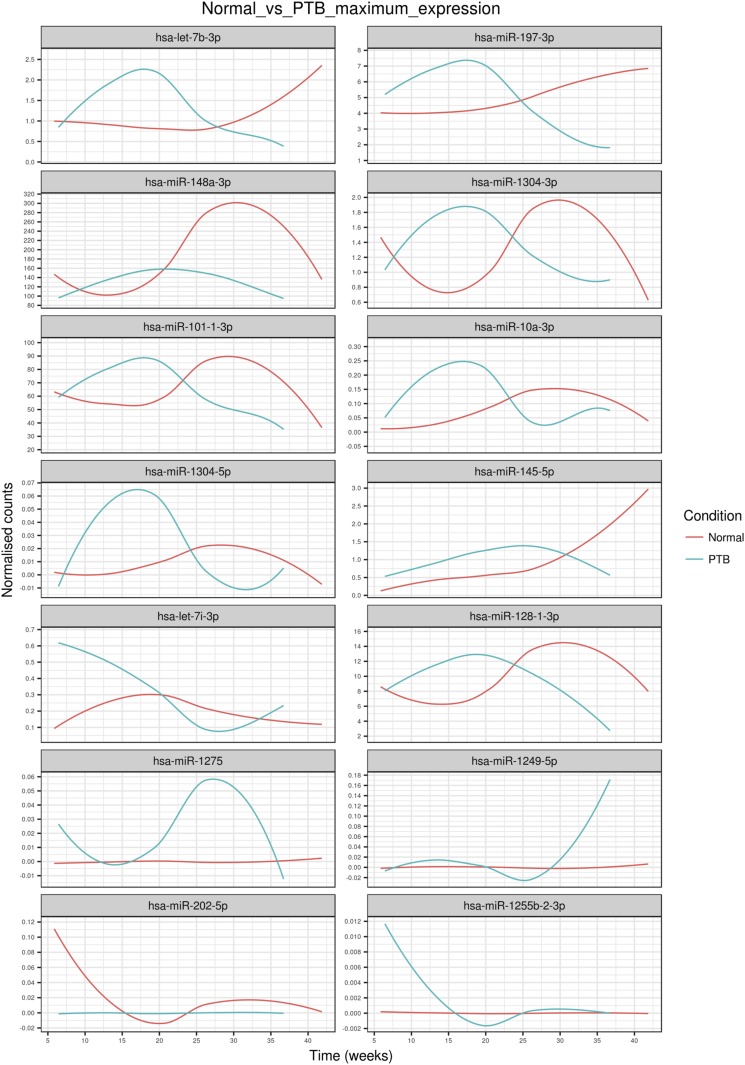
Highest expressed miRNAs in each cluster identified using linear mixed modeling when comparing normal pregnancies to PTB pregnancies across gestation. Linear mixed modeling and circular cladogram analysis when comparing normal pregnancies to PTB pregnancies across gestation were divided into 14 clusters. The highest expressed miRNA from each cluster was extracted and color coded based on the cluster of origin. Clusters are in ascending order [corresponding to (A)–(N) in Fig. 6] starting from the top of the figure, reading from left to right and continuing onto the next line of graphs below. Red indicates normal pregnancies whereas blue indicates PTB pregnancies.

A total of 173 miRNAs were found to significantly change (*P* < 0.05) across gestation for normal compared with PTB pregnancies. Hierarchical clustering analysis of the average miRNA expression profiles across gestation revealed a variety of trends (Fig. 6). Specifically, trends that had the largest difference in expression within the first trimester comparing normal to PTB pregnancies are clusters I, M, and N (I = 8, M = 15, N = 43); trends that had the largest difference in expression within the second trimester comparing normal to PTB pregnancies are clusters A, D, F, G, J, and K (A = 14, D = 5, F = 5, G = 8, J = 17, K = 13); and trends that had the largest difference in expression within the third trimester comparing normal to PTB pregnancies are clusters B, C, E, H, and L (B = 5, C = 9, E = 12, H = 6, L = 13). miRNAs with the largest maximum expression in each cluster are as follows (Fig. 7; Table 4): A, hsa-let-7b-3p; B, hsa-miR-197-3p; C, hsa-miR-148a-3p; D, hsa-miR-1304-3p; E, hsa-miR-101-1-3p; F, hsa-miR-10a-3p; G, hsa-miR-1304-5p; H, hsa-miR-145-5p; I, hsa-let-7i-3p; J, hsa-miR-128-1-3p; K, hsa-miR-1275; L, hsa-miR-1249-5p; M, hsa-miR-202-5p; and N, hsa-miR-1255b-2-3p.

**Table 4.** Significant miRNAs That Change Across Gestation for Normal Versus PTB Pregnancies

Condition	miRNA	Gestational Age With Minimum Expression	Minimum Expression (Normalized Counts)	Gestational Age With Maximum Expression	Maximum Expression (Normalized Counts)	*P* Value	Letter
Normal	hsa-let-7b-3p	19.000	0.405	40.429	2.590	0.044	A
PTB	hsa-let-7b-3p	36.286	0.077	12.857	2.627	0.044	A
Normal	hsa-miR-1249-3p	18.429	0.071	6.143	0.211	0.006	A
PTB	hsa-miR-1249-3p	36.714	−0.011	26.143	0.375	0.006	A
Normal	hsa-miR-1260a	19.000	1.028	6.143	3.592	0.012	A
PTB	hsa-miR-1260a	36.714	0.118	19.143	2.901	0.012	A
Normal	hsa-miR-1260b	41.857	1.489	5.857	4.403	0.001	A
PTB	hsa-miR-1260b	36.714	0.078	18.714	4.841	0.001	A
Normal	hsa-miR-130b-5p	18.571	0.251	6.286	1.578	0.013	A
PTB	hsa-miR-130b-5p	36.714	−0.008	26.571	1.287	0.013	A
Normal	hsa-miR-18a-3p	18.857	0.143	6.143	1.584	0.025	A
PTB	hsa-miR-18a-3p	36.571	0.033	26.143	1.081	0.025	A
Normal	hsa-miR-222-3p	41.857	6.562	5.857	18.303	0.039	A
PTB	hsa-miR-222-3p	36.286	2.457	19.429	19.327	0.039	A
Normal	hsa-miR-22-5p	18.714	0.067	5.857	0.227	0.010	A
PTB	hsa-miR-22-5p	6.571	0.026	18.571	0.370	0.010	A
Normal	hsa-miR-3677-3p	18.714	0.039	41.857	0.164	0.041	A
PTB	hsa-miR-3677-3p	36.286	−0.008	12.143	0.144	0.041	A
Normal	hsa-miR-4433b-5p	19.000	1.083	6.429	19.855	0.020	A
PTB	hsa-miR-4433b-5p	36.000	0.569	6.571	18.526	0.020	A
Normal	hsa-miR-4446-3p	19.143	1.216	5.857	7.518	0.033	A
PTB	hsa-miR-4446-3p	36.714	1.288	26.286	5.254	0.033	A
Normal	hsa-miR-483-3p	19.000	0.188	40.429	0.543	0.017	A
PTB	hsa-miR-483-3p	6.571	−0.012	19.143	0.674	0.017	A
Normal	hsa-miR-494-3p	41.857	0.019	5.857	0.177	0.009	A
PTB	hsa-miR-494-3p	36.714	−0.025	19.286	0.248	0.009	A
Normal	hsa-miR-5010-5p	19.000	0.520	6.143	2.483	0.011	A
PTB	hsa-miR-5010-5p	36.000	0.349	19.143	2.647	0.011	A
Normal	hsa-miR-197-3p	19.000	2.458	37.000	8.277	0.001	B
PTB	hsa-miR-197-3p	36.000	0.812	18.571	7.698	0.001	B
Normal	hsa-miR-24-1-3p	13.857	3.735	39.000	14.270	0.029	B
PTB	hsa-miR-24-1-3p	36.571	2.000	12.857	11.039	0.029	B
Normal	hsa-miR-24-2-3p	19.000	3.081	39.000	14.547	0.029	B
PTB	hsa-miR-24-2-3p	36.571	2.855	12.857	10.512	0.029	B
Normal	hsa-miR-339-5p	6.143	0.216	40.857	1.027	0.034	B
PTB	hsa-miR-339-5p	32.571	0.087	18.571	0.716	0.034	B
Normal	hsa-miR-424-3p	6.143	1.693	41.857	4.630	0.009	B
PTB	hsa-miR-424-3p	36.571	0.644	18.714	3.890	0.009	B
Normal	hsa-miR-148a-3p	10.286	79.632	27.429	312.515	0.028	C
PTB	hsa-miR-148a-3p	7.143	85.944	18.571	165.603	0.028	C
Normal	hsa-miR-152-3p	10.286	0.358	28.143	2.730	0.044	C
PTB	hsa-miR-152-3p	36.000	0.390	13.000	1.897	0.044	C
Normal	hsa-miR-223-3p	13.857	7.682	27.000	89.305	0.012	C
PTB	hsa-miR-223-3p	36.000	16.825	18.571	44.779	0.012	C
Normal	hsa-miR-3615	13.571	2.862	28.143	9.084	0.029	C
PTB	hsa-miR-3615	34.429	2.152	19.429	5.248	0.029	C
Normal	hsa-miR-550a-1-5p	13.000	0.142	28.143	0.585	0.004	C
PTB	hsa-miR-550a-1-5p	34.429	0.036	12.857	0.512	0.004	C
Normal	hsa-miR-550a-1-5p_var2	12.429	0.127	28.143	0.569	0.004	C
PTB	hsa-miR-550a-1-5p_var2	35.000	0.026	12.143	0.476	0.004	C
Normal	hsa-miR-550a-2-5p	13.000	0.133	27.000	0.580	0.004	C
PTB	hsa-miR-550a-2-5p	35.857	0.042	13.000	0.488	0.004	C
Normal	hsa-miR-550a-2-5p_var2	12.000	0.117	27.000	0.574	0.004	C
PTB	hsa-miR-550a-2-5p_var2	36.714	0.036	12.143	0.500	0.004	C
Normal	hsa-miR-550a-3-5p	13.571	0.112	28.143	0.462	0.005	C
PTB	hsa-miR-550a-3-5p	36.429	0.005	19.143	0.349	0.005	C
Normal	hsa-miR-1304-3p	41.857	0.675	28.143	2.003	0.018	D
PTB	hsa-miR-1304-3p	36.000	0.850	18.714	1.882	0.018	D
Normal	hsa-miR-361-5p	19.000	1.221	37.000	3.102	0.020	D
PTB	hsa-miR-361-5p	36.429	1.080	19.143	3.191	0.020	D
Normal	hsa-miR-423-3p	13.571	33.079	27.429	119.044	0.009	D
PTB	hsa-miR-423-3p	36.286	30.309	18.571	88.296	0.009	D
Normal	hsa-miR-92a-1-3p	19.000	1247.304	27.000	3712.668	0.002	D
PTB	hsa-miR-92a-1-3p	36.714	1202.248	19.429	3587.438	0.002	D
Normal	hsa-miR-92a-2-3p	19.000	1142.639	27.000	3732.389	0.002	D
PTB	hsa-miR-92a-2-3p	36.714	1241.370	19.429	3553.979	0.002	D
Normal	hsa-miR-101-1-3p	39.571	16.739	27.000	114.873	0.021	E
PTB	hsa-miR-101-1-3p	36.000	21.077	19.429	94.615	0.021	E
Normal	hsa-miR-101-2-3p	40.857	20.180	27.000	118.287	0.020	E
PTB	hsa-miR-101-2-3p	36.000	17.958	18.571	93.111	0.020	E
Normal	hsa-miR-103a-1-3p	40.857	38.378	27.000	247.984	0.046	E
PTB	hsa-miR-103a-1-3p	36.000	62.859	9.714	220.993	0.046	E
Normal	hsa-miR-103a-2-3p	40.857	45.190	27.000	243.098	0.046	E
PTB	hsa-miR-103a-2-3p	34.429	67.586	9.714	219.811	0.046	E
Normal	hsa-miR-106b-3p	40.857	5.819	27.000	24.471	0.041	E
PTB	hsa-miR-106b-3p	36.000	2.176	19.429	19.745	0.041	E
Normal	hsa-miR-107	40.857	44.043	27.000	236.691	0.047	E
PTB	hsa-miR-107	36.000	50.350	9.714	224.599	0.047	E
Normal	hsa-miR-140-3p	19.000	15.453	27.000	45.277	0.013	E
PTB	hsa-miR-140-3p	36.000	7.497	18.571	41.822	0.013	E
Normal	hsa-miR-16-2-3p	39.571	5.010	26.286	20.848	0.033	E
PTB	hsa-miR-16-2-3p	36.714	4.330	7.857	19.259	0.033	E
Normal	hsa-miR-29c-3p	13.571	3.852	27.000	14.952	0.040	E
PTB	hsa-miR-29c-3p	26.857	3.551	18.571	11.390	0.040	E
Normal	hsa-miR-342-3p	39.571	5.199	27.000	12.179	0.026	E
PTB	hsa-miR-342-3p	36.000	0.799	18.571	13.442	0.026	E
Normal	hsa-miR-425-5p	39.571	14.436	27.000	45.780	0.011	E
PTB	hsa-miR-425-5p	36.000	5.925	18.571	38.338	0.011	E
Normal	hsa-miR-502-3p	39.571	1.636	27.000	7.404	0.022	E
PTB	hsa-miR-502-3p	36.000	0.962	18.571	6.580	0.022	E
Normal	hsa-miR-10a-3p	11.286	−0.030	26.286	0.197	0.014	F
PTB	hsa-miR-10a-3p	26.857	−0.033	13.000	0.335	0.014	F
Normal	hsa-miR-379-5p	41.857	−0.003	26.429	0.007	0.026	F
PTB	hsa-miR-379-5p	32.571	−0.007	13.000	0.027	0.026	F
Normal	hsa-miR-524-3p	8.571	−0.006	28.143	0.059	0.029	F
PTB	hsa-miR-524-3p	26.857	−0.010	12.857	0.118	0.029	F
Normal	hsa-miR-525-3p	8.571	−0.004	28.143	0.029	0.020	F
PTB	hsa-miR-525-3p	32.571	−0.001	13.000	0.076	0.020	F
Normal	hsa-miR-548j-3p	13.714	−0.012	27.429	0.062	0.019	F
PTB	hsa-miR-548j-3p	26.714	−0.015	12.857	0.067	0.019	F
Normal	hsa-miR-1304-5p	41.857	−0.005	27.429	0.026	0.049	G
PTB	hsa-miR-1304-5p	32.571	−0.012	18.571	0.064	0.049	G
Normal	hsa-miR-138-1-3p	41.857	−0.006	28.143	0.019	0.025	G
PTB	hsa-miR-138-1-3p	6.571	−0.028	18.571	0.066	0.025	G
Normal	hsa-miR-3918	27.000	−0.002	8.571	0.005	0.048	G
PTB	hsa-miR-3918	6.429	−0.010	19.143	0.038	0.048	G
Normal	hsa-miR-4419a	39.000	−0.002	12.429	0.002	0.042	G
PTB	hsa-miR-4419a	6.429	−0.011	18.714	0.021	0.042	G
Normal	hsa-miR-4742-3p	41.857	0.024	5.857	0.130	0.032	G
PTB	hsa-miR-4742-3p	6.429	−0.033	18.571	0.170	0.032	G
Normal	hsa-miR-491-5p	41.857	−0.007	27.000	0.021	0.037	G
PTB	hsa-miR-491-5p	6.429	−0.021	18.714	0.051	0.037	G
Normal	hsa-miR-5189-3p	20.286	0.006	5.857	0.145	0.007	G
PTB	hsa-miR-5189-3p	36.429	−0.005	18.714	0.172	0.007	G
Normal	hsa-miR-6516-5p	41.857	−0.002	6.286	0.011	0.031	G
PTB	hsa-miR-6516-5p	6.429	−0.014	18.714	0.032	0.031	G
Normal	hsa-miR-145-5p	6.286	0.115	41.857	2.864	0.023	H
PTB	hsa-miR-145-5p	6.429	0.468	26.857	1.474	0.023	H
Normal	hsa-miR-371b-5p	8.571	0.070	41.857	1.473	0.041	H
PTB	hsa-miR-371b-5p	6.571	0.161	19.143	0.623	0.041	H
Normal	hsa-miR-377-5p	13.571	−0.005	41.857	0.154	0.047	H
PTB	hsa-miR-377-5p	26.857	−0.008	19.429	0.159	0.047	H
Normal	hsa-miR-3928-3p	13.571	−0.004	40.857	0.125	0.049	H
PTB	hsa-miR-3928-3p	34.429	−0.006	13.000	0.090	0.049	H
Normal	hsa-miR-6791-5p	6.429	−0.011	40.429	0.096	0.028	H
PTB	hsa-miR-6791-5p	36.000	−0.014	9.714	0.091	0.028	H
Normal	hsa-miR-6803-3p	27.000	0.014	40.857	0.130	0.024	H
PTB	hsa-miR-6803-3p	36.714	−0.027	6.429	0.140	0.024	H
Normal	hsa-let-7i-3p	39.571	0.055	12.857	0.343	0.050	I
PTB	hsa-let-7i-3p	26.714	0.030	7.857	0.734	0.050	I
Normal	hsa-miR-1908-3p	41.857	0.002	5.857	0.161	0.022	I
PTB	hsa-miR-1908-3p	32.571	0.015	9.714	0.300	0.022	I
Normal	hsa-miR-520a-3p	6.143	0.120	28.143	0.847	0.022	I
PTB	hsa-miR-520a-3p	32.571	0.051	10.571	1.184	0.022	I
Normal	hsa-miR-539-3p	12.429	0.011	28.143	0.218	0.010	I
PTB	hsa-miR-539-3p	36.429	−0.025	12.857	0.357	0.010	I
Normal	hsa-miR-589-3p	19.000	0.040	6.143	0.151	0.019	I
PTB	hsa-miR-589-3p	35.286	0.018	12.143	0.258	0.019	I
Normal	hsa-miR-6837-3p	11.286	0.001	26.429	0.141	0.021	I
PTB	hsa-miR-6837-3p	36.571	−0.003	18.714	0.181	0.021	I
Normal	hsa-miR-6852-5p	12.571	0.976	27.000	1.882	0.014	I
PTB	hsa-miR-6852-5p	36.000	0.466	18.571	3.062	0.014	I
Normal	hsa-miR-760	6.429	0.013	41.857	0.151	0.003	I
PTB	hsa-miR-760	32.571	0.007	6.429	0.435	0.003	I
Normal	hsa-miR-128-1-3p	13.857	3.453	26.286	15.183	0.013	J
PTB	hsa-miR-128-1-3p	36.714	1.818	19.429	13.765	0.013	J
Normal	hsa-miR-128-2-3p	13.286	2.890	26.286	14.693	0.013	J
PTB	hsa-miR-128-2-3p	36.714	1.788	19.429	12.515	0.013	J
Normal	hsa-miR-1285-1-3p	39.571	0.142	27.000	2.477	0.036	J
PTB	hsa-miR-1285-1-3p	32.571	0.392	7.857	3.242	0.036	J
Normal	hsa-miR-1285-2-3p	39.571	0.217	12.000	2.464	0.032	J
PTB	hsa-miR-1285-2-3p	36.000	0.418	7.857	3.108	0.032	J
Normal	hsa-miR-140-5p	13.286	0.028	27.429	0.328	0.024	J
PTB	hsa-miR-140-5p	36.000	−0.017	13.000	0.412	0.024	J
Normal	hsa-miR-199a-1-5p	40.857	0.619	27.000	4.821	0.039	J
PTB	hsa-miR-199a-1-5p	36.000	−0.002	19.143	4.775	0.039	J
Normal	hsa-miR-199a-2-5p	39.571	0.743	27.000	4.759	0.039	J
PTB	hsa-miR-199a-2-5p	36.000	−0.010	19.143	5.143	0.039	J
Normal	hsa-miR-224-5p	19.000	0.159	27.000	2.291	0.009	J
PTB	hsa-miR-224-5p	36.714	0.002	18.714	2.244	0.009	J
Normal	hsa-miR-25-3p	39.571	47.155	38.857	343.596	0.007	J
PTB	hsa-miR-25-3p	36.714	−13.802	7.857	349.212	0.007	J
Normal	hsa-miR-28-5p	40.857	1.226	6.143	9.605	0.038	J
PTB	hsa-miR-28-5p	32.571	2.047	18.571	5.961	0.038	J
Normal	hsa-miR-330-3p	40.857	0.170	6.429	1.881	0.046	J
PTB	hsa-miR-330-3p	36.000	0.032	19.429	1.609	0.046	J
Normal	hsa-miR-410-3p	19.000	0.577	28.143	11.516	0.015	J
PTB	hsa-miR-410-3p	36.714	1.621	19.429	7.912	0.015	J
Normal	hsa-miR-433-3p	13.571	0.029	40.857	0.404	0.014	J
PTB	hsa-miR-433-3p	36.714	0.022	13.000	0.493	0.014	J
Normal	hsa-miR-484	19.000	16.538	27.000	41.184	0.050	J
PTB	hsa-miR-484	36.714	−2.373	26.571	70.214	0.050	J
Normal	hsa-miR-485-5p	12.143	0.172	5.857	0.796	0.017	J
PTB	hsa-miR-485-5p	34.429	0.121	19.429	1.094	0.017	J
Normal	hsa-miR-6735-5p	40.429	−0.009	20.571	0.225	0.024	J
PTB	hsa-miR-6735-5p	36.571	−0.045	18.571	0.217	0.024	J
Normal	hsa-miR-98-3p	13.857	0.167	27.000	0.835	0.003	J
PTB	hsa-miR-98-3p	36.714	0.060	19.429	1.090	0.003	J
Normal	hsa-miR-1275	12.143	−0.004	40.857	0.006	0.048	K
PTB	hsa-miR-1275	36.714	−0.010	26.857	0.061	0.048	K
Normal	hsa-miR-1298-5p	40.857	−0.002	5.857	0.005	0.027	K
PTB	hsa-miR-1298-5p	13.000	−0.007	26.571	0.032	0.027	K
Normal	hsa-miR-3138	6.429	−0.028	20.286	0.103	0.012	K
PTB	hsa-miR-3138	36.571	−0.137	26.571	0.641	0.012	K
Normal	hsa-miR-32-3p	6.429	−0.006	26.143	0.014	0.013	K
PTB	hsa-miR-32-3p	36.714	−0.029	26.571	0.142	0.013	K
Normal	hsa-miR-3689a-3p	28.143	−0.009	12.429	0.022	0.002	K
PTB	hsa-miR-3689a-3p	36.714	−0.053	26.857	0.241	0.002	K
Normal	hsa-miR-3689a-5p	19.000	−0.001	20.571	0.070	0.026	K
PTB	hsa-miR-3689a-5p	36.571	−0.069	26.857	0.218	0.026	K
Normal	hsa-miR-3689b-5p	12.857	−0.005	20.571	0.075	0.026	K
PTB	hsa-miR-3689b-5p	7.857	−0.061	26.857	0.212	0.026	K
Normal	hsa-miR-3689e	12.857	−0.006	12.429	0.062	0.026	K
PTB	hsa-miR-3689e	36.571	−0.056	26.857	0.225	0.026	K
Normal	hsa-miR-409-3p	13.571	5.563	27.000	17.099	0.008	K
PTB	hsa-miR-409-3p	36.714	−9.655	26.571	71.852	0.008	K
Normal	hsa-miR-4467	19.000	−0.006	5.857	0.059	0.001	K
PTB	hsa-miR-4467	6.429	−0.010	26.714	0.075	0.001	K
Normal	hsa-miR-597-5p	13.857	−0.002	27.000	0.005	0.014	K
PTB	hsa-miR-597-5p	36.714	−0.008	6.429	0.032	0.014	K
Normal	hsa-miR-6750-3p	28.143	−0.002	19.143	0.004	0.014	K
PTB	hsa-miR-6750-3p	36.714	−0.006	26.857	0.029	0.014	K
Normal	hsa-miR-889-5p	28.143	−0.001	12.857	0.005	0.019	K
PTB	hsa-miR-889-5p	36.429	−0.006	26.714	0.027	0.019	K
Normal	hsa-miR-1249-5p	28.143	−0.009	41.857	0.013	0.020	L
PTB	hsa-miR-1249-5p	26.286	−0.027	36.714	0.176	0.020	L
Normal	hsa-miR-141-3p	41.857	−3.057	27.000	8.385	0.039	L
PTB	hsa-miR-141-3p	26.857	−4.227	36.714	53.676	0.039	L
Normal	hsa-miR-146b-3p	19.143	−1.544	26.429	3.242	0.041	L
PTB	hsa-miR-146b-3p	26.571	−3.755	36.571	50.654	0.041	L
Normal	hsa-miR-193a-5p	41.857	−0.010	28.143	0.061	0.032	L
PTB	hsa-miR-193a-5p	26.143	−0.033	36.714	0.189	0.032	L
Normal	hsa-miR-200a-3p	6.429	−1.486	28.143	7.882	0.040	L
PTB	hsa-miR-200a-3p	26.286	−3.358	36.571	50.665	0.040	L
Normal	hsa-miR-376a-1-5p	41.857	−0.013	6.143	0.089	0.035	L
PTB	hsa-miR-376a-1-5p	26.857	−0.024	36.571	0.178	0.035	L
Normal	hsa-miR-382-5p	20.571	−0.047	6.429	0.347	0.037	L
PTB	hsa-miR-382-5p	26.143	−0.023	36.571	0.600	0.037	L
Normal	hsa-miR-3936	12.571	−8.814	12.000	9.239	0.015	L
PTB	hsa-miR-3936	26.857	−28.825	36.714	171.652	0.015	L
Normal	hsa-miR-4447	38.857	−11.147	27.000	6.753	0.015	L
PTB	hsa-miR-4447	26.857	−24.998	36.714	166.559	0.015	L
Normal	hsa-miR-4516	28.143	−3.132	6.286	2.609	0.045	L
PTB	hsa-miR-4516	26.286	−3.764	36.571	50.392	0.045	L
Normal	hsa-miR-4695-5p	11.286	−0.011	26.571	0.013	0.045	L
PTB	hsa-miR-4695-5p	6.429	−0.024	36.714	0.174	0.045	L
Normal	hsa-miR-6769b-5p	19.143	−0.014	6.143	0.050	0.018	L
PTB	hsa-miR-6769b-5p	26.714	−0.026	36.714	0.168	0.018	L
Normal	hsa-miR-7976	37.714	−0.013	26.429	0.060	0.030	L
PTB	hsa-miR-7976	6.571	−0.034	36.571	0.397	0.030	L
Normal	hsa-miR-202-5p	20.286	−0.020	5.857	0.151	0.030	M
PTB	hsa-miR-202-5p	36.714	−0.004	36.286	0.004	0.030	M
Normal	hsa-miR-3656	41.857	−0.034	5.857	0.309	0.006	M
PTB	hsa-miR-3656	35.000	−0.030	13.000	0.244	0.006	M
Normal	hsa-miR-4535	19.000	−0.008	6.429	0.052	0.027	M
PTB	hsa-miR-4535	6.571	−0.008	18.714	0.027	0.027	M
Normal	hsa-miR-4650-1-3p	20.286	−0.002	6.286	0.024	0.046	M
PTB	hsa-miR-4650-1-3p	6.571	−0.002	18.714	0.010	0.046	M
Normal	hsa-miR-4650-2-3p	20.571	−0.001	6.143	0.024	0.046	M
PTB	hsa-miR-4650-2-3p	6.571	−0.003	18.571	0.011	0.046	M
Normal	hsa-miR-4688	19.143	−0.004	5.857	0.054	0.046	M
PTB	hsa-miR-4688	6.429	−0.003	26.143	0.032	0.046	M
Normal	hsa-miR-4717-3p	20.286	−0.002	6.429	0.013	0.047	M
PTB	hsa-miR-4717-3p	6.571	−0.002	19.286	0.003	0.047	M
Normal	hsa-miR-4789-3p	19.143	−0.010	5.857	0.049	0.032	M
PTB	hsa-miR-4789-3p	6.429	−0.010	19.143	0.024	0.032	M
Normal	hsa-miR-4798-5p	39.000	−0.080	5.857	1.121	0.048	M
PTB	hsa-miR-4798-5p	36.571	−0.117	26.143	0.506	0.048	M
Normal	hsa-miR-545-5p	19.000	−0.021	5.857	0.190	0.040	M
PTB	hsa-miR-545-5p	36.429	−0.011	6.429	0.062	0.040	M
Normal	hsa-miR-6859-1-3p	18.857	−0.007	5.857	0.053	0.017	M
PTB	hsa-miR-6859-1-3p	6.429	−0.003	36.714	0.010	0.017	M
Normal	hsa-miR-6859-2-3p	18.714	−0.007	5.857	0.054	0.017	M
PTB	hsa-miR-6859-2-3p	6.571	−0.001	36.286	0.008	0.017	M
Normal	hsa-miR-6859-3-3p	19.000	−0.007	5.857	0.054	0.017	M
PTB	hsa-miR-6859-3-3p	6.571	−0.002	36.571	0.010	0.017	M
Normal	hsa-miR-6859-4-3p	18.857	−0.007	5.857	0.054	0.017	M
PTB	hsa-miR-6859-4-3p	6.429	−0.002	36.714	0.009	0.017	M
Normal	hsa-miR-7856-5p	19.143	−0.008	6.286	0.043	0.028	M
PTB	hsa-miR-7856-5p	6.429	−0.005	18.571	0.027	0.028	M
Normal	hsa-miR-1255b-2-3p	20.571	−0.001	6.429	0.001	0.014	N
PTB	hsa-miR-1255b-2-3p	19.143	−0.002	6.429	0.012	0.014	N
Normal	hsa-miR-129-1-3p	12.571	−0.001	19.143	0.000	0.014	N
PTB	hsa-miR-129-1-3p	19.429	−0.002	6.429	0.012	0.014	N
Normal	hsa-miR-129-2-3p	6.286	0.000	6.429	0.000	0.014	N
PTB	hsa-miR-129-2-3p	19.143	−0.002	6.429	0.012	0.014	N
Normal	hsa-miR-1343-3p	41.857	−0.002	5.857	0.007	0.029	N
PTB	hsa-miR-1343-3p	36.571	−0.001	6.429	0.033	0.029	N
Normal	hsa-miR-147b	12.429	−0.004	39.000	0.003	0.042	N
PTB	hsa-miR-147b	32.571	−0.015	7.857	0.109	0.042	N
Normal	hsa-miR-15a-3p	41.857	−0.001	13.571	0.002	0.031	N
PTB	hsa-miR-15a-3p	19.429	−0.003	6.571	0.023	0.031	N
Normal	hsa-miR-2277-3p	6.286	−0.014	19.143	0.028	0.001	N
PTB	hsa-miR-2277-3p	18.714	−0.037	6.429	0.254	0.001	N
Normal	hsa-miR-301b-5p	41.857	−0.002	28.143	0.004	0.043	N
PTB	hsa-miR-301b-5p	18.714	−0.001	6.429	0.021	0.043	N
Normal	hsa-miR-3116-1	38.857	−0.001	37.000	0.001	0.014	N
PTB	hsa-miR-3116-1	19.429	−0.002	6.429	0.011	0.014	N
Normal	hsa-miR-3116-2	39.571	0.000	10.286	0.000	0.014	N
PTB	hsa-miR-3116-2	19.429	−0.002	6.429	0.011	0.014	N
Normal	hsa-miR-3184-3p	27.429	−0.001	13.000	0.002	0.046	N
PTB	hsa-miR-3184-3p	19.429	−0.003	6.571	0.044	0.046	N
Normal	hsa-miR-337-3p	19.000	−0.003	5.857	0.094	0.017	N
PTB	hsa-miR-337-3p	36.714	−0.024	6.571	0.323	0.017	N
Normal	hsa-miR-3665	6.429	−0.011	13.714	0.010	0.004	N
PTB	hsa-miR-3665	19.286	−0.034	6.571	0.212	0.004	N
Normal	hsa-miR-379-3p	41.857	−0.046	27.429	0.286	0.004	N
PTB	hsa-miR-379-3p	36.714	−0.044	6.571	0.606	0.004	N
Normal	hsa-miR-3908	26.429	−0.001	6.286	0.004	0.007	N
PTB	hsa-miR-3908	19.286	−0.007	6.429	0.038	0.007	N
Normal	hsa-miR-3942-3p	10.286	−0.003	27.429	0.003	0.006	N
PTB	hsa-miR-3942-3p	18.571	−0.009	6.571	0.059	0.006	N
Normal	hsa-miR-411-3p	41.857	−0.046	27.429	0.274	0.004	N
PTB	hsa-miR-411-3p	36.714	−0.050	6.571	0.616	0.004	N
Normal	hsa-miR-4301	41.857	−0.001	5.857	0.002	0.013	N
PTB	hsa-miR-4301	35.286	−0.002	6.429	0.032	0.013	N
Normal	hsa-miR-4318	27.000	−0.001	10.286	0.003	0.031	N
PTB	hsa-miR-4318	19.286	−0.003	6.429	0.023	0.031	N
Normal	hsa-miR-433-5p	41.857	−0.001	6.143	0.001	0.046	N
PTB	hsa-miR-433-5p	19.429	−0.002	6.571	0.021	0.046	N
Normal	hsa-miR-4502	19.143	−0.002	13.571	0.001	0.040	N
PTB	hsa-miR-4502	19.286	−0.002	6.429	0.022	0.040	N
Normal	hsa-miR-4522	20.286	0.000	37.714	0.001	0.014	N
PTB	hsa-miR-4522	19.286	−0.002	6.429	0.011	0.014	N
Normal	hsa-miR-4529-3p	26.429	−0.001	6.286	0.001	0.039	N
PTB	hsa-miR-4529-3p	19.429	−0.002	6.429	0.012	0.039	N
Normal	hsa-miR-4530	13.286	−0.021	41.857	0.015	0.004	N
PTB	hsa-miR-4530	18.714	−0.068	6.429	0.407	0.004	N
Normal	hsa-miR-4640-3p	12.143	0.000	38.857	0.001	0.014	N
PTB	hsa-miR-4640-3p	19.143	−0.002	6.571	0.011	0.014	N
Normal	hsa-miR-4709-5p	6.143	−0.003	18.714	0.004	0.008	N
PTB	hsa-miR-4709-5p	18.714	−0.007	6.429	0.038	0.008	N
Normal	hsa-miR-4769-5p	12.429	−0.001	39.000	0.001	0.003	N
PTB	hsa-miR-4769-5p	19.429	−0.003	6.429	0.019	0.003	N
Normal	hsa-miR-500a-5p	26.429	−0.010	11.286	0.019	0.007	N
PTB	hsa-miR-500a-5p	19.286	−0.067	6.429	0.421	0.007	N
Normal	hsa-miR-500b-5p	41.857	−0.015	11.286	0.026	0.006	N
PTB	hsa-miR-500b-5p	19.143	−0.069	6.429	0.430	0.006	N
Normal	hsa-miR-5698	6.286	−0.010	39.000	0.007	0.009	N
PTB	hsa-miR-5698	19.286	−0.014	6.429	0.208	0.009	N
Normal	hsa-miR-572	37.714	−0.001	12.857	0.001	0.003	N
PTB	hsa-miR-572	19.143	−0.003	6.429	0.019	0.003	N
Normal	hsa-miR-579-3p	5.857	−0.001	19.143	0.002	0.015	N
PTB	hsa-miR-579-3p	19.429	−0.003	6.571	0.023	0.015	N
Normal	hsa-miR-586	19.000	−0.002	12.571	0.001	0.003	N
PTB	hsa-miR-586	18.571	−0.007	6.429	0.038	0.003	N
Normal	hsa-miR-655-3p	20.571	−0.001	19.143	0.001	0.014	N
PTB	hsa-miR-655-3p	19.429	−0.003	6.429	0.024	0.014	N
Normal	hsa-miR-6759-5p	6.286	−0.001	12.857	0.001	0.003	N
PTB	hsa-miR-6759-5p	19.286	−0.003	6.429	0.020	0.003	N
Normal	hsa-miR-6783-3p	39.571	−0.003	5.857	0.003	0.005	N
PTB	hsa-miR-6783-3p	19.143	−0.008	6.429	0.082	0.005	N
Normal	hsa-miR-6797-5p	8.286	−0.001	19.143	0.001	0.003	N
PTB	hsa-miR-6797-5p	19.429	−0.004	6.429	0.019	0.003	N
Normal	hsa-miR-6798-5p	12.143	−0.001	20.571	0.001	0.003	N
PTB	hsa-miR-6798-5p	19.429	−0.004	6.571	0.019	0.003	N
Normal	hsa-miR-6802-5p	41.857	−0.015	28.143	0.040	0.032	N
PTB	hsa-miR-6802-5p	19.429	−0.009	6.429	0.105	0.032	N
Normal	hsa-miR-6838-3p	12.571	0.000	10.286	0.001	0.014	N
PTB	hsa-miR-6838-3p	18.571	−0.002	6.429	0.011	0.014	N
Normal	hsa-miR-6856-5p	40.857	−0.001	28.143	0.002	0.024	N
PTB	hsa-miR-6856-5p	19.429	−0.002	6.429	0.012	0.024	N
Normal	hsa-miR-8079	5.857	−0.001	12.429	0.000	0.046	N
PTB	hsa-miR-8079	19.429	−0.002	6.429	0.022	0.046	N
Normal	hsa-miR-933	26.571	−0.001	13.714	0.003	0.028	N
PTB	hsa-miR-933	19.429	−0.004	6.429	0.023	0.028	N

### Pathway analysis and gene target and gene ontology prediction

**Figure 8.**
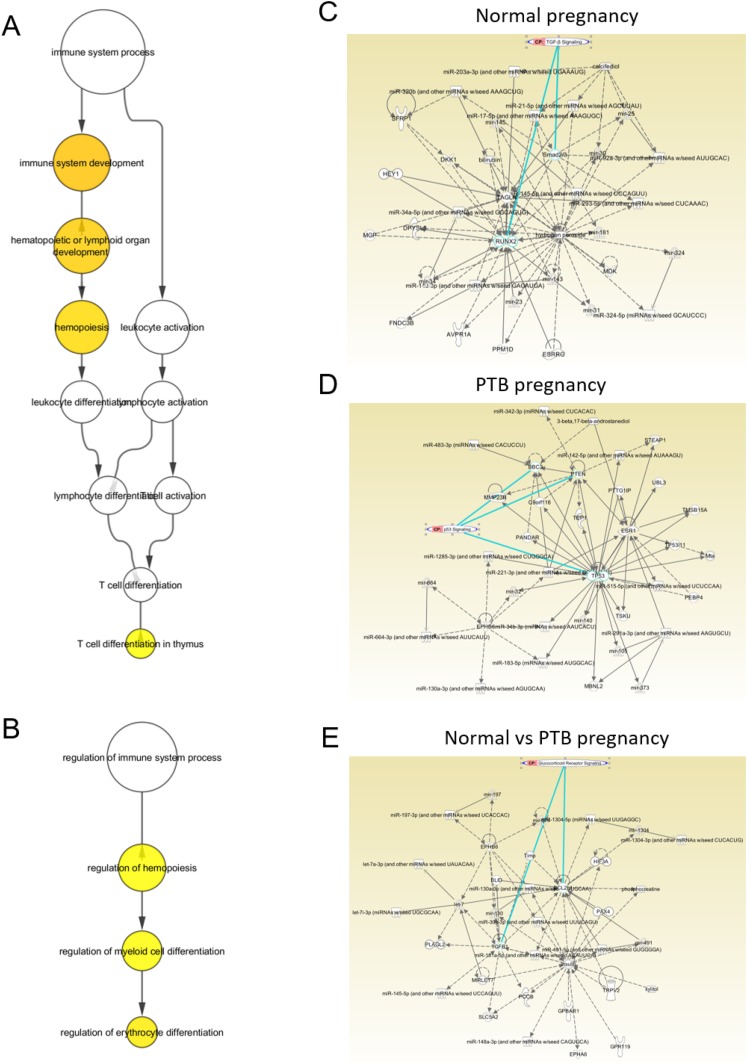
miRNA gene target and gene ontology analysis for the highest abundant miRNAs per cluster in each condition. Gene target analysis for significant and highest abundant miRNAs in each cluster for normal, PTB, and normal vs PTB pregnancies across gestation are shown. Gene ontology analysis for these gene targets showed enrichment for immune systems process in (A) normal and (B) PTB pregnancies. The yellow circles indicate statistically significant gene ontologies (*P* value <0.05), while the change of color from yellow to orange indicates increasing statistical significance. Bioinformatics analysis on the largest maximum expression miRNA profile in (C) term, (D) PTB, and (E) term vs PTB show differences in the miRNA profile between these groups target signaling pathways (circled in green) associated with (C) TGF-*β* signalling, (D) p53, and (E) glucocorticoid receptor signalling.

Gene target and pathway analyses of the largest maximum expression exosomal miRNAs that were revealed to be differentially expressed in term, PTB, and term vs PTB across gestation were analyzed using *in silico* tools, including the CyTargetLinker application in Cytoscape, as well as Ingenuity pathway analysis (IPA). In term, PTB, and term vs PTB pregnancies, the largest maximum expression miRNAs were linked to 645, 1677, and 686 target genes, respectively, and these gene targets were represented in at least two miRNA databases. The target genes of the differentially expressed miRNAs were subjected to gene ontology analysis using the BiNGO application in Cytoscape that identified a total of 1000, 1172, and 995 gene ontology terms, respectively, being regulated by these miRNAs. Within this gene ontology network, pathways regulating the immune system process were enriched (*P* < 0.05) in term and PTB pregnancies (Fig. 8A and 8B). Then, we performed a bioinformatics analysis on the largest maximum expression miRNA profile in term, PTB, and term vs PTB analysis, and our data establish that the differences in the miRNA profile between these groups target the signaling pathways associated with TGF-*β* signaling, p53, and glucocorticoid receptor signaling, respectively (Fig. 8C–8E).

## Discussion

The aim of this study was to characterize serial changes in the exosomal miRNA concentration present in maternal circulation across gestation in term and PTB pregnancies. We implemented an innovative approach using linear mixed modeling to determine exosomal miRNA expression as a function of gestational age in term and PTB pregnancies. The data obtained identified exosomal miRNA changes specific to term and PTB as a function of the gestational age during pregnancy.

### Exosomal miRNA changes in normal pregnancies

miRNA clusters that are differentially regulated are functionally linked to various physiologic and pathologic conditions during pregnancy. These functions include regulation of cell growth (miR-3200) (25), control of cell transition factors in female reproductive tract by IGF receptor 1 and TGF-*β* receptor 2 functions (miR-3200-5p) (26), anomalous placentation (miR-320a) (27), epithelial mesenchymal transition involved in tissue remodeling (miR-25-3p) (28), gestational hypertension, preeclampsia, and intrauterine growth restriction (miR-143-3p) (29), cell proliferation and migration (miR129-1 cluster) (30), oocyte aging and embryogenesis (miR-203a-3p) (29, 31), cell proliferation and migration (miR-324-5p) (32), MAPK function and induction of cell cycle arrest as seen in senescent uterine cells (miR129-1 cluster) (30), and immune responses and inflammatory reactions (miR-6769b-5p) (33).

miRNAs collectively represent TGF-*β* signaling during various stages of gestation ending in term deliveries (34, 35). TGF-*β* is an anti-inflammatory cytokine, and ongoing work in the Menon laboratory suggests that they can contribute to epithelial mesenchymal transitions of amnion epithelial cells to maintain fetal membrane homeostasis. In placenta, TGF-*β*1 and IGFBP-3 have been shown to signal through TGF-*β* receptors to influence human cytotrophoblast proliferation (36). Similarly, TGF-*β*1 upregulates connexin43-mediated gap junctional intercellular communication required for human trophoblast differentiation (37). Therefore, miRNA profiling in exosomes during normal gestation and parturition indicates that TGF-*β–*mediated tissue homeostasis (regulates cellular proliferation and transitions) is required for pregnancy maintenance.

### Exosomal miRNA changes in PTB

Examination of differentially expressed exosomal miRNA in PTB samples provided more distinct clusters than for normal birth. A summary of functional roles already reported for many of the miRNAs identified in exosomes from PTB suggests that miRNAs are functionally linked to cell cycle regulation (let-7a-2-5p) (38, 39), MAPK kinase kinases and inhibition of cell growth (miR-520a-3p) (40), cellular transitions and inflammation (miR-520a-3p and miR-483-3p) (41, 42), placental oxidative stress response and impairment of mitochondrial function (miR-130b-3p and miR-4433b-3p) (43, 44), macrophage programming contributing to inflammatory and oxidative stress damages (miR-142-5p) (45), preeclampsia (miR-342-3p) (46, 47), as well as placental dysfunction and impaired fetal growth due to folate deficiency (miR-222-3p) (48).

### PTB

One of the key pathways affected in PTB (as determined in our IPA analysis) is a p53-mediated mechanism (49). The function of p53 is overtly represented in exosomes during gestation ending in PTB compared with normal term birth. p53 is a well-reported tumor suppressor protein that is implicated in placental and decidual cell senescence and fetal membrane apoptosis (50). Overrepresentation of the p53 pathway in exosomes during pregnancies ending in PTB suggests disturbances in normal cell cycle indicative of cellular senescence and/or programmed cell death.

IPA analysis of differentially expressed miRNAs represented glucocorticoid signaling as one of the key pathways implicated in PTB. Several reports by Zakar *et al.* (51, 52) and Zakar and Olson (53) have shown the effect of glucocorticoids on prostaglandin production (uterotonin) as well as their role in organ maturation and fetal growth. In most mammalian species, increased concentrations of glucocorticoids are evident in the maternal and fetal circulations and amniotic fluid prior to the onset of labor. Glucocorticoids directly increase fetal placental prostaglandin production, and they indirectly increase prostaglandin production by maternal uterine tissues through the stimulation of placental estradiol synthesis (54, 55). Thus, exosomal miRNA representation of glucocorticoid signaling implicates untimely activation of this signaling in PTB during early gestation compared with those ending in normal term deliveries (56, 57).

The strengths of this study are longitudinal sampling at four separate time points from a well-defined cohort of subjects and technological approaches to isolate and characterize exosomal miRNAs. This study precisely is a hypothesis-generation work, and the pathways and networks identified in this study still need further testing using *in vitro* cell culture models or animal models. We have correlated the networks and pathways to pregnancy whenever data were available in the literature and or suggested potential functions based on the reported functions in other systems. Another important point about this study is that we profiled the miRNAs associated with the total circulating exosomes present in maternal plasma across gestation, and the origin of these exosomes (*i.e.*, placenta) was not identified. Owing to technical difficulties, miRNA quantity that can be obtained from the fetal portion of maternal plasma (∼15% to 20%) is not sufficient to yield a fetal-only miRNA signature. Thus, the miRNA profile is an indication of an overall change in fetal as well as maternal tissues and is not restricted to either fetal or uterine tissues. Regardless, the exosome miRNA profile is unique at different trimesters, at term and preterm, and it differs substantially between pregnancies ending up with preterm and term births. Irrespective of origin of exosomes, these are indicative of overall physiologic changes in fetomaternal tissues.

In summary, the exosomal miRNAs showing maximum expression for each trend of change were associated with inflammation and cellular movement for term pregnancy. Alternatively, the top molecular and cellular functions targeted by the top exosomal miRNAs in PTB were cell-to-cell signaling and interaction, cellular growth and proliferation, and cellular development. Comparative analysis between the exosomal miRNA in term and PTB reveals that main differences are in cell morphology and cellular development. The data presented in this study establish that circulating exosomes carry a specific set of miRNAs as a function of the gestational age in term pregnancy, and that the circulating exosomal miRNA profile changes in PTB pregnancies compared with normal term deliveries. We posit that exosomes may contribute to the inflammatory state and metabolic changes associated with pregnancy by the specific incorporation of miRNAs, which might be delivered to maternal cells to support the normal maternal physiological changes during gestation, a phenomenon dysregulated in PTB pregnancies.

## Abbreviations:

IPA: Ingenuity pathway analysis
PTB: preterm birth
